# MicroRNA 3′ ends shorten during adolescent brain maturation

**DOI:** 10.3389/fnmol.2023.1168695

**Published:** 2023-04-14

**Authors:** Kristen T. Thomas, Anaïs Vermare, Suzannah O. Egleston, Yong-Dong Wang, Ashutosh Mishra, Tong Lin, Junmin Peng, Stanislav S. Zakharenko

**Affiliations:** ^1^Department of Developmental Neurobiology, St. Jude Children’s Research Hospital, Memphis, TN, United States; ^2^Department of Cell and Molecular Biology, St. Jude Children’s Research Hospital, Memphis, TN, United States; ^3^Center for Proteomics and Metabolomics, St. Jude Children’s Research Hospital, Memphis, TN, United States; ^4^Department of Biostatistics, St. Jude Children’s Research Hospital, Memphis, TN, United States; ^5^Department of Structural Biology, St. Jude Children’s Research Hospital, Memphis, TN, United States

**Keywords:** microRNA, adolescence, neurodevelopment, brain maturation, isomiRs, miR-338-3p, RNA-seq, quantitative proteomics

## Abstract

MicroRNA (miRNA) dysregulation is well-documented in psychiatric disease, but miRNA dynamics remain poorly understood during adolescent and early adult brain maturation, when symptoms often first appear. Here, we use RNA sequencing to examine miRNAs and their mRNA targets in cortex and hippocampus from early-, mid-, and late-adolescent and adult mice. Furthermore, we use quantitative proteomics by tandem mass tag mass spectrometry (TMT-MS) to examine protein dynamics in cortex from the same subjects. We found that ~25% of miRNAs’ 3′ ends shorten with age due to increased 3′ trimming and decreased U tailing. Particularly, shorter but functionally competent isoforms (isomiRs) of miR-338-3p increase up to 10-fold during adolescence and only in brain. MiRNAs that undergo 3′ shortening exhibit stronger negative correlations with targets that decrease with age and stronger positive correlations with targets that increase with age, than miRNAs with stable 3′ ends. Increased 3′ shortening with age was also observed in available mouse and human miRNA-seq data sets, and stronger correlations between miRNAs that undergo shortening and their mRNA targets were observed in two of the three available data sets. We conclude that age-associated miRNA 3′ shortening is a well-conserved feature of postnatal brain maturation.

## 1. Introduction

MicroRNA (miRNA) dysregulation is well-documented in human subjects and animal models of psychiatric disease. MiRNAs are small noncoding RNAs, typically 17–25 nucleotides in length, that bind to mRNAs containing complementary sequences and promote degradation and/or inhibit translation of the mRNA target. MiRNA levels are altered in postmortem brain samples from human subjects with schizophrenia (Perkins et al., 2007; Beveridge et al., 2008, 2010; Kim et al., 2010; Moreau et al., 2011; Miller et al., 2012; Liu et al., 2018; Hu et al., 2019), bipolar disorder (Bavamian et al., 2015; Azevedo et al., 2016), major depressive disorder (Smalheiser et al., 2012; Lopez et al., 2014; Roy et al., 2017; Yoshino et al., 2021), substance use disorders (Lim et al., 2021; Santos-Bezerra et al., 2021), and other psychiatric disorders. Genetic studies implicate a strong role for miRNAs in psychiatric disease risk and symptoms (Cummings et al., 2013; Duan et al., 2014; Mühleisen et al., 2014; Ripke et al., 2014; Forstner et al., 2015; Kandratsenka et al., 2018; Mahmoudi et al., 2021; Tielke et al., 2022). Additionally, studies in animal models implicate miRNAs in disease etiology and suggest that they may be viable therapeutic targets for the treatment of psychiatric diseases (Chun et al., 2017; Li et al., 2021; Liang et al., 2022).

The prevalence of psychiatric disorders dramatically increases during adolescence and early adulthood (Solmi et al., 2022). Adolescent and post-adolescent brain maturation are characterized by widespread changes in myelination, dopaminergic signaling, GABAergic signaling, synapse numbers, synaptic plasticity, and immune function (Thomas and Zakharenko, 2021). These processes are driven by complex changes in gene expression, both transcriptionally and post-transcriptionally, and miRNAs have been implicated in all of these processes within the mammalian brain (Thomas and Zakharenko, 2021). However, miRNA studies in mouse models of psychiatric disease are frequently conducted in adult mice and fail to consider age as a variable, and few studies have examined miRNA dynamics during typical adolescent brain maturation.

MiRNA levels in the mammalian brain are highly dynamic across the lifespan, but existing studies have reported contradictory results regarding specific changes in miRNAs levels with age. Several studies report that miRNA levels decline between childhood and adulthood (Beveridge et al., 2014). Others report that they increase (Miska et al., 2004; Moreau et al., 2013). Additional studies report complex waves of miRNA expression during different stages of development (Eda et al., 2011; Ziats and Rennert, 2014; Hu et al., 2019). These discrepancies may be due in part to biological differences between studies, e.g., the brain regions examined, the age distributions of the subjects, and the species of the subjects. However, differences in the methods of miRNA detection might also contribute to differences in these studies’ findings.

MiRNAs are most commonly detected by sequencing (miRNA-seq), microarrays, or reverse transcription-quantitative PCR (RT-qPCR). Each of these methods has strengths and weaknesses (Git et al., 2010; Cloonan et al., 2011; Leshkowitz et al., 2013; Dard-Dascot et al., 2018; Godoy et al., 2019; Wright et al., 2019). For example, RT-qPCR is often considered the “gold standard” of miRNA detection due to its high sensitivity and ability to detect small changes in miRNA levels, yet RT-qPCR probes widely differ in their sensitivity to both miRNA sequence variants, or “isomiRs,” and to miRNA modifications, e.g., 2′-O-methylation. Microarrays profile hundreds of miRNAs with high speed and at a relatively low cost. However, microarrays may struggle to detect some isomiRs or distinguish miRNAs with similar sequences, and they have a limited quantitative range. MiRNA-seq distinguishes sequence variants unambiguously in theory, but it is susceptible to sequence detection bias, batch effects, and sequencing errors. Therefore, validating results using multiple methods for detecting miRNAs is critical.

In the present study, we undertake a detailed examination of miRNAs and their targets in the mouse cortex and hippocampus during adolescence and early adulthood. In addition to changes in miRNA levels, we observed widespread shortening of miRNA 3′ ends during adolescent brain maturation. Age-associated 3′ shortening appears to be conserved across multiple brain regions, developmental stages, and species (mice and humans). Although its functional consequences remain unclear, miRNAs that exhibit 3′ shortening with age also exhibit stronger correlations with mRNA and protein targets involved in neurodevelopment, synaptic function, and posttranscriptional gene expression.

## 2. Results

### 2.1. MiRNA levels and miR-338-3p 3′ trimmed isomiRs increase with age

Mouse adolescence begins at weaning at approximately postnatal day (P) 20–22, extends into mid-adolescence with the onset of puberty at ~P40, and ends as mice reach full sexual maturity and enter early adulthood at ~P60 (Supplementary Figure S1A; Spear, 2000). Mature adulthood is reached at ~P120 (Spear, 2000). We conducted pilot studies to identify miRNAs that might serve as positive controls for additional experiments, identify timepoints when transitions in miRNA levels occurred, and determine whether any global changes in miRNA levels might occur with age. Previous work demonstrated that miR-338-3p decreases in mouse auditory thalamus (the ventral portion of the medial geniculate body, MGv) between 2 months (mo) and 4 mo of age (Chun et al., 2017). Using RT-qPCR, we found that miR-338-3p levels declined between adolescence and adulthood in mouse cortex, hippocampus, and auditory thalamus (Supplementary Figures S1B,C). Expression patterns during adolescence varied slightly across tissues. MiR-338-3p levels remained stable after 4 mo.

The normalization strategy strongly influences the results of all differential expression (DE) analysis methods. Normalization in RT-qPCR typically depends on one or a few “housekeeping” RNAs that are assumed to remain stable with age, whereas typical normalization strategies utilized in microarray and RNA-seq experiments assume that the total level of RNA is constant across samples. In order to determine if the total level of miRNA might change with age, we examined previously published (Chun et al., 2017) microarray data for wildtype mice 2 and 4 mo of age and normalized the data to spike-in RNAs that were added during sample preparation. Using this normalization strategy, we found that most miRNAs decreased with age in all brain tissues examined (Supplementary Figures S1D,E). Notably, miR-338-3p significantly decreased with age in all data sets. However, this normalization strategy depends heavily on the assumption that endogenous RNA levels were equal prior to spike-in addition and on the accurate measurement of only a few spike-in probes.

**Figure 1:**
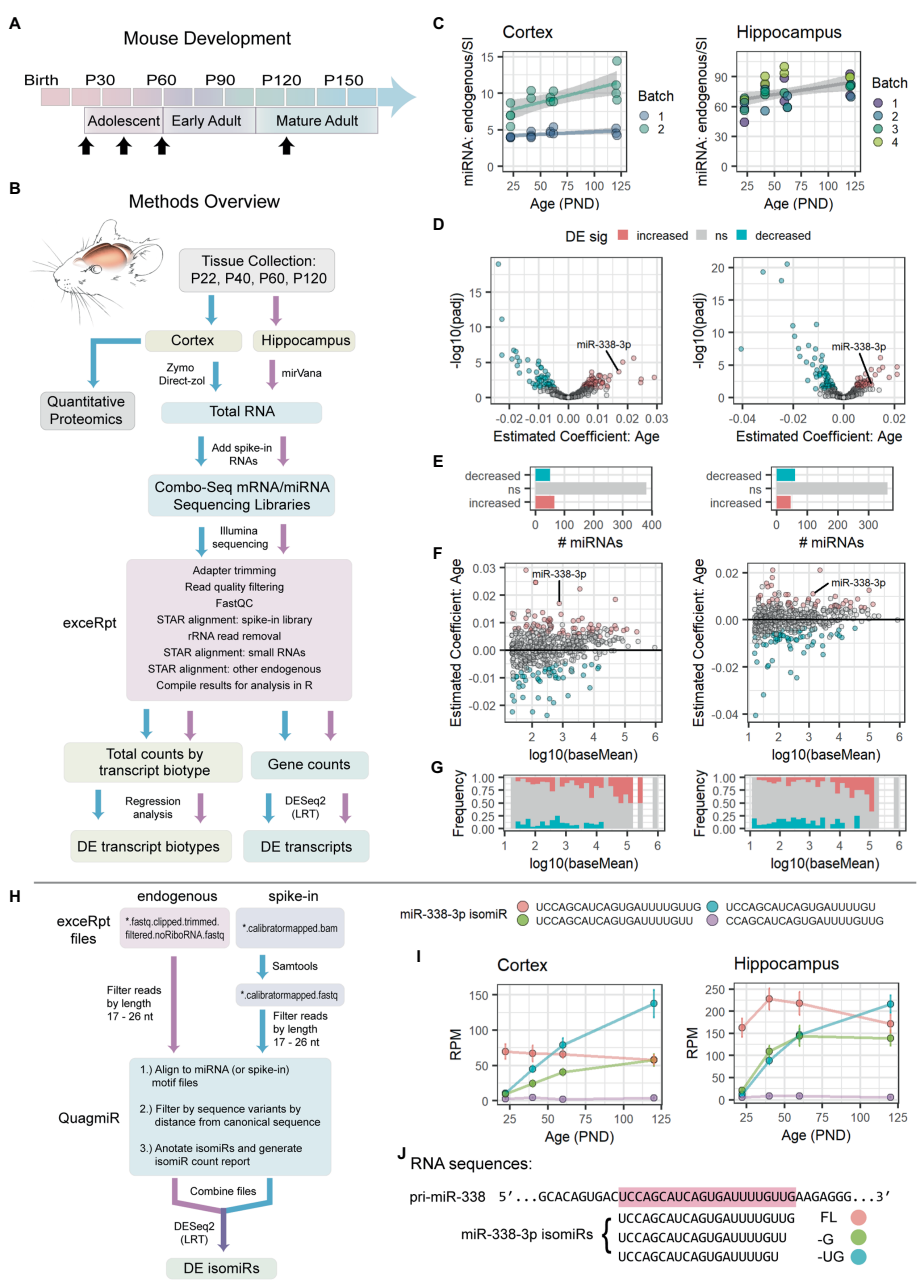
miR-338-3p levels and 3′ trimming increase during adolescence. **(A)** Timeline of mouse postnatal development. Timepoints for RNA-seq and quantitative proteomics experiments are indicated with black arrows: P22, P40, P60, P120 (±2 days). **(B)** Overview of RNA-seq and quantitative proteomics experiments. Mouse cortex and hippocampus were collected at the time points indicated in **(A)**. Approximately half of each cortical sample was used for quantitative proteomics experiments. Total RNA was extracted from remaining tissue and used to prepare combined mRNA/miRNA sequencing libraries (Combo-Seq). Large and small spike-in RNAs were added to each sample prior to library preparation. Following Illumina sequencing, data were analyzed FIGURE 1 (Continued)using exceRpt and DESeq2. **(C)** Total miRNA levels increase in cortex (left) and hippocampus (right) between P22 and P120. For each sample, total endogenous miRNA reads were normalized to total miRNA spike-in reads obtained *via* Combo-Seq. Significance was assessed *via* linear regression modeling: miRNA endogenous to spike-in ratio ~ PND + Batch + Sex. Cortex: p(Age) = 0.00116, Estimate (Age) = 0.023556, Adjusted *R*^2^ = 0.8083. Hippocampus: p(Age) = 0.00264, Estimate (Age) = 0.1730, Adjusted *R*^2^ = 0.3989. Trend lines were fit using the geom_smooth() function in R, with method set to “lm.” Trend lines were fit by batch for the Cortex data set due to significant effect of batch in the regression model: p(Batch) = 4.45e-10 (Batch), Estimate (Batch) = 4.668375. **(D–G)** Summary of differential expression (DE) analysis results for endogenous miRNAs in Combo-Seq data sets from cortex (left) and hippocampus (right). DE was assessed by likelihood ratio test (LRT) in DESeq2. MiRNA read mapping was performed in exceRpt. **(D)** Volcano plots summarizing adjusted value of *p* and age coefficient data. **(E)** Bar plots summarizing the number of miRNAs in each DE category. Overall, a similar number of miRNAs undergo a significant increase or decrease with age in each data set. **(F)** MA plots summarizing age coefficients and mean normalized read counts (baseMean). Positive age coefficients indicate miRNAs that undergo an overall increase between P22 and P120, while negative coefficients indicate miRNAs that decrease with age. **(G)** Frequency of DE miRNAs varies by miRNA level. Highly abundant miRNAs are more likely to increase with age. **(H)** Overview of isomiR analysis strategy. Fastq files were filtered by length. Reads were then mapped to miRNA motifs, filtered by distance from the canonical (miRBase) miRNA sequence, and summarized in an isomiR report using QuagmiR. IsomiR DE analysis was then performed *via* LRT in DESeq2. **(I)** Summary data (mean ± standard error) for the 4 most abundant miR-338-3p isomiRs in cortex (left) and hippocampus (right) between P22 and P120. Association between age and isomiR levels was assessed by LRT in DESeq2. UCCAGCAUCAGUGAUUUUGUUG (cortex): *p* = 0.2103878, Estimate (Age) = −0.003359707. UCCAGCAUCAGUGAUUUUGUU (cortex): *p* = 2.010535e-04, Estimate (Age) = 0.022727726. UCCAGCAUCAGUGAUUUUGU (cortex): *p* = 1.959909e-08, Estimate (Age) = 0.031463167. CCAGCAUCAGUGAUUUUGUUG (cortex): *p* = 0.8085618, Estimate (Age) = 0.002651315. UCCAGCAUCAGUGAUUUUGUUG (hippocampus): *p* = 0.7757617, Estimate (Age) = −0.000946411. UCCAGCAUCAGUGAUUUUGUU (hippocampus): *p* = 0.01248705, Estimate (Age) = 0.016375420. UCCAGCAUCAGUGAUUUUGU (hippocampus): *p* = 5.439813e-06, Estimate (Age) = 0.028297607. CCAGCAUCAGUGAUUUUGUUG (hippocampus): *p* = 0.6533541, Estimate (Age) = −0.002867399. All the *p*-values listed above were adjusted for multiple comparisons. RPM, reads per million miRNA reads. **(J)** Alignment of miR-338-3p isomiRs with the mouse pri-miR-338 sequence. The canonical, or “full length” (FL), sequence is highlighted in red. Two 3′ trimmed isomiRs, −G and −UG, are also shown. See Supplementary Figure S1 for miR-338-3p RT-qPCR data, miRNA microarray data, and Combo-Seq sample ages, PCA results and miR-338-3p normalized counts. See Supplementary Figure S2 for analysis of spike-in RNAs. PND: postnatal days.

Based on these pilot studies, we decided to examine mouse hippocampus and cortex at P22, P40, P60, and P120 ± 2 (Figure 1A). Tissues were collected from ~8 mice of both sexes at each time point (Supplementary Figures S1F,I). RNA isolations, library preparations, and sequencing were conducted independently for the cortex and hippocampus. MiR-338-3p was identified as a positive control that reliably declined across multiple detection methods and multiple tissues in our pilot studies.

We hypothesized that overall miRNA levels decline during early adulthood in mouse brain. To test this hypothesis and determine whether a similar phenomenon also occurs during adolescence, we chose a combined mRNA/miRNA sequencing (Combo-Seq) strategy, which allows detection of mRNAs, lincRNAs, miRNAs, and other small noncoding RNAs from a single library preparation (Figure 1B, see Table 1 for glossary of terms). After RNA isolation and prior to library preparation, ERCC spike-in RNAs (i.e., large spike-in RNAs or mRNA-like spike-in RNAs) and “miRNA-like” small spike-in RNAs were added to each sample in equal amounts. MiRNA levels could then be normalized to spike-in RNAs or a combination of spike-in and endogenous RNAs. In addition, mRNA target levels could then be measured within the sample library preparation. Because miRNAs can influence mRNA translation with or without affecting target mRNA levels, we also collected cortical tissue for quantitative proteomics from the same animals. We predicted that this approach would provide a robust measurement of overall miRNA levels during brain maturation and valuable insights into miRNA target biology at the mRNA and protein levels.

**Table 1:** Glossary of terms.

Term	Definition
3′ end regression analysis (3′ERA)	A linear regression-based method to assess changes in the length of the 3′ end of a miRNA
3′ extension	Increase in the length of a miRNA’s 3′ end due to decreased 3′ trimming and/or increased 3′ tailing
3′ shortening	Reduction in the length of a miRNA’s 3′ end due to increased 3′ trimming and/or increased 3′ tailing
3′ tailing	Addition of nucleotides to the 3′ end of a miRNA
3′ trimming	Removal of nucleotides from the 3′ end of a miRNA, typically by an exoribonuclease
Canonical miRNA sequence	The sequence listed in miRBase for a miRNA
Combo-Seq	An approach for sequencing mRNAs, miRNAs, and other noncoding RNAs using a single library preparation
isomiR	MiRNA sequence variant, i.e., a miRNA isoform
Large spike-in RNA	An exogenous RNA that resembles an mRNA and is added to a sample prior to RNA-seq library preparation
Small spike-in RNA	An exogenous RNA that resembles a miRNA and is added to a sample prior to RNA-seq library preparation
miR-338-3p FL	miR-338-3p canonical isomiR: 5′-UCCAGCAUCAGUGAUUUUGUUG-3′
miR-338-3p -G	miR-338-3p 3′ trimmed isomiR: 5′-UCCAGCAUCAGUGAUUUUGUU-3′
miR-338-3p -UG	miR-338-3p 3′ trimmed isomiR: 5′-UCCAGCAUCAGUGAUUUUGU-3′

However, contrary to our hypothesis, we found that total miRNA levels significantly increased with brain maturation in both the cortex and hippocampus (Figure 1C). We then performed DE analysis using all endogenous and spike-in RNA counts for data normalization. As expected, samples clustered by age when we performed principal component analysis (PCA) using endogenous miRNA reads (Supplementary Figures S1G,J). However, contrary to our pilot study results, DE analysis of individual miRNAs revealed that miR-338-3p significantly increased with age (Figures 1D,F and Supplementary Figures S1H,K). The increase in total miRNA levels appeared to be driven by an increase in a subset of highly abundant miRNAs (Figure 1G), as only a small subset of miRNAs significantly increased with age (Figure 1E).

As expected, most spike-in RNAs were correctly classified as not DE with age (Supplementary Figures S2A–D). However, an unexpectedly high number of small spike-in RNAs appeared to decrease with age in libraries prepared from cortex, suggesting that the results shown in Figures 1D–G underestimate the number of endogenous miRNAs that increase with age in the cortex. Correlation analysis and hierarchical clustering revealed that spike-in reads were highly correlated across samples and that samples clustered by library batch, not by subject age (Supplementary Figures S2E,F). Consistent with previous reports (Leshkowitz et al., 2013; Godoy et al., 2019; Wright et al., 2019), spike-in reads were poorly correlated with the *a priori* known levels of individual small spike-in RNAs (Supplementary Figures S2G,H). Therefore, Combo-Seq does not reliably measure the relative levels of individual miRNAs within each sample but can reliably assess differences between samples.

Contrary to our hypothesis and the results of our pilot studies, miR-338-3p and overall miRNA levels increased with brain maturation in our Combo-Seq experiments. We therefore sought an explanation for this discrepancy. Although RT-qPCR and microarrays are highly quantitative, they both often struggle to distinguish between miRNAs with similar sequences and, conversely, to reliably detect isomiRs, i.e., miRNA sequence variants. We hypothesized that isomiR variance might affect our results. We therefore modified our analysis pipeline to perform DE on individual isomiRs (Figure 1H).

When we examined miR-338-3p isomiRs, we found that they dramatically differed in their expression patterns during adolescent and early adult brain maturation (Figure 1I). While the canonical form of miR-383-3p (ending in …GUUG) declines between adolescence and adulthood, two isomiRs increased ~8–10-fold between P22 and P120. These isomiRs differ from the canonical form in the 3′ end: one is trimmed by 1 nucleotide (ending in …GUU), while the other is trimmed by 2 (ending in …GU) (Figure 1J). We will refer to these isomiRs as miR-338-3p FL (of “full length”), −G, and −UG, respectively. By P120, the −G and −UG isomiRs together constitute a majority of the miR-338-3p isomiR reads detected (Figure 1I), suggesting that miR-338-3p undergoes an increase in 3′ trimming during brain maturation.

### 2.2. MiR-338-3p 3′ trimmed isomiRs are detectable by RT-qPCR, associate with AGO proteins, and inhibit targets

**Figure 2:**
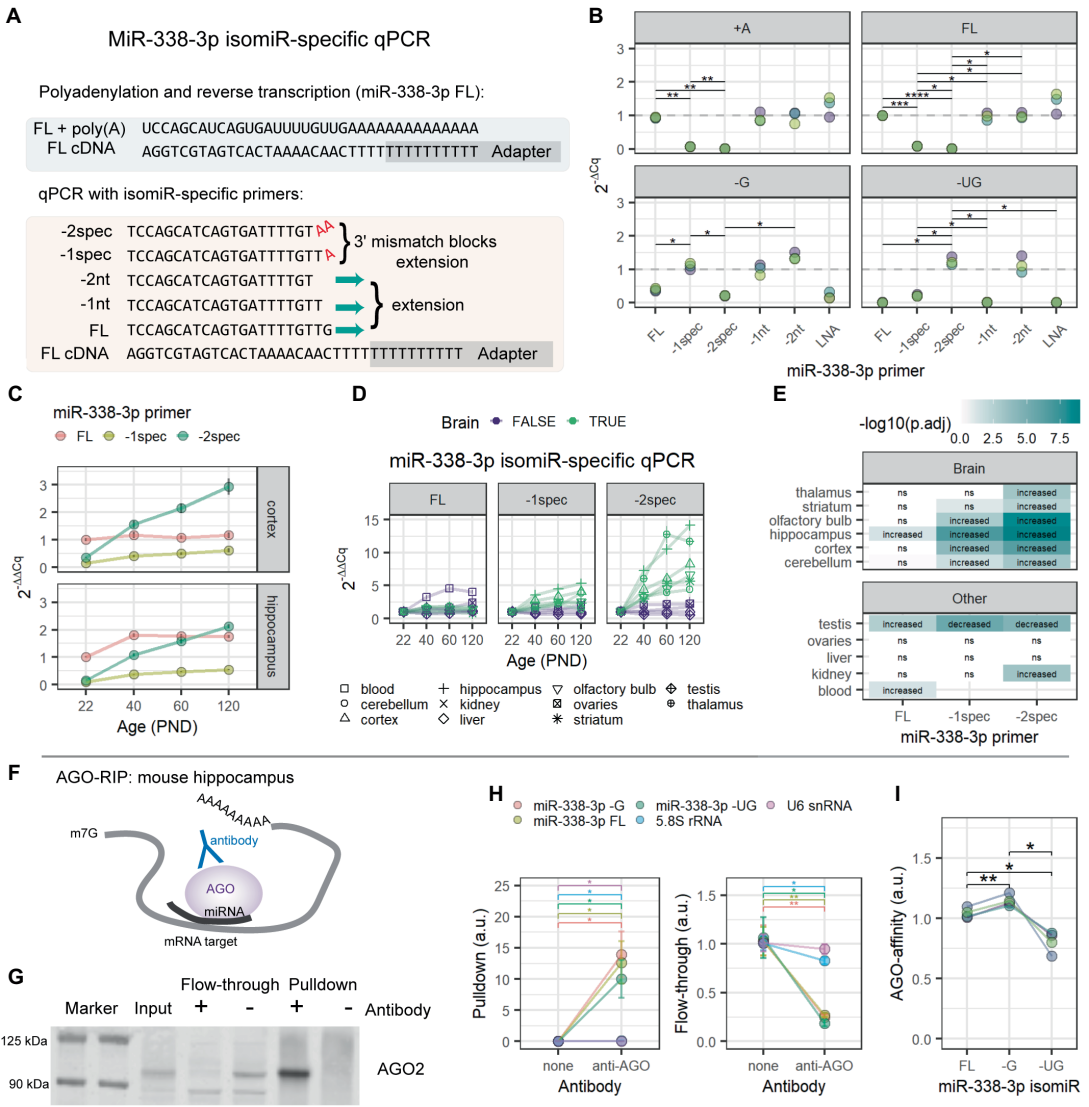
MiR-338-3p 3′ trimmed isomiRs increase with age and associate with AGO proteins in mouse hippocampus. **(A)** Overview of isomiR-specific RT-qPCR assays for miR-338-3p. MiRNAs are first poly(A)-tailed. Reverse transcription is performed with a poly(T) primer containing a universal adapter sequence. RT-qPCR is then performed with a forward primer targeting the isomiR of interest and a universal reverse primer matching the adapter sequence. As shown, the miR-338-3p FL primer fully anneals to cDNA made from the FL isomiR. However, −G and −UG isomiRs are excluded due to mismatches at the 3′ end of primer binding, which prevents efficient RT-qPCR amplification. However, the -1spec and -2spec primers fully anneal to the −G and −UG isomiRs, respectively, while excluding longer forms by adding terminal A nucleotides to each primer that match the poly(A) tail added prior to reverse transcription. **(B)** MiR-338-3p isomiR detection by 3′ isomiR-specific RT-qPCR assays. RNA oligomers were commercially synthesized and used as templates for the assays shown in **(A)**. All Cq values were normalized to the mean Cq value for miR-338-3p FL isomiR detection by the miR-338-3p FL primer. *N* = 3. Two-way ANOVA: p(isomiR) = 4.96e-04, p(primer) = 2.69e-05, p(interactive) = 9.54e-21. Holm-adjusted *p*-values for pairwise comparisons: *p < 0.05, **p < 0.01, ***p < 0.001, ****p < 0.0001. **(C)** MiR-338-3p 3′ trimmed isomiRs increased between P22 and P120. IsomiRs were detected by isomiR-specific RT-qPCR assay described in **(A)**. All Cq values were normalized within tissue to the mean Cq value for miR-338-3p FL isomiR detection by the miR-338-3p FL primer. **(D)** Increases in miR-338-3p 3′ trimmed isomiRs between P22 and P120 might be brain-specific. The -UG isomiR increases with age in all brain tissues observed, but not in non-brain tissues, other than a small increase in kidney. Similarly, the -G isomiR increases with age in most brain tissues, except thalamus and striatum, but none of the non-brain tissues. **(E)** Statistical results for data in **(D)**. Data were analyzed by linear regression modeling within each tissue: isomiR ~ Age + Sex. *P*-values were adjusted for multiple comparisons using the FDR method. “Increased”: p(Age) < 0.05 & Estimate (Age) > 0. “Decreased”: p(Age) < 0.05 and Estimate (Age) < 0. “ns”: *p* > 0.05. **(F)** Overview of AGO-RIP experiments in **(G–I)**. AGO-miRNA-mRNA target complexes were pulled down with an AGO-specific antibody. **(G)** Western blot validation of AGO-IP protein fractions. AGO2 was depleted in the flow-through and enriched in the pulldown fraction when an AGO-specific antibody was used for IP. In the bead only (no AGO antibody) condition, AGO2 was not detectable in the pulldown fraction and remained in the flow-through. **(H)** qPCR detection of RNA in pulldown (left) and flow-through fractions (right) from AGO-RIP experiments in mouse hippocampus. MiR-338-3p FL, −G, and −UG isomiRs associate with AGO proteins with high affinity and were significantly depleted in flow-through. Significant, but lower-affinity, associations were also observed between AGO and 5.8S rRNA and U6 snRNA. *N* = 4 biological replicates. Two-way ANOVA (pulldown): p(RNA) = 0.000247, p(antibody) = 0.036000, p(interactive) = 0.000249. Two-way ANOVA (flow-through): p(RNA) = 1.00e-03, p(antibody) = 8.00e-03, p(interactive) = 3.98e-05. Holm adjusted *p*-values for pairwise comparisons: *p < 0.05, **p < 0.01. **(I)** Relative AGO affinities of the miR-338-3p FL, −G, and −UG as detected by RT-qPCR from the pulldown fraction of AGO-IPs shown in **(H)**. The −G isomiR appears to have a slightly higher affinity than the other isomiRs, whereas the −UG isomiR has the lowest affinity. One-way repeated measures ANOVA: *p* = 0.018. Holm adjusted *p*-values for pairwise comparisons: *p < 0.05, **p < 0.01. See Supplementary Figure S3 for functional assays examining miR-338-3p isomiR mimics in Neuro-2a cells.

We hypothesized that this increase in miR-338-3p 3′ trimming might interfere with detection by RT-qPCR. In RT-qPCR, detection is highly sensitive to mismatches at the 3′ end of each primer (Lefever et al., 2013). We used this sensitivity to design miR-338-3p primers specific to each of the 3 most abundant miR-338-3p isomiRs detected in our Combo-Seq data sets (Figure 2A). This is similar to the approach previously described by Nejad et al., 2018. As expected, primers designed to target the canonical form of miR-338-3p detected the canonical form as well as longer isomiRs (Figure 2B). Modification of the 3′ end of the primer blocked detection of the canonical form and allowed detection of the 3′ trimmed isomiRs. We applied this isomiR-specific RT-qPCR assay to the samples previously used for Combo-Seq. Our primers replicated our Combo-Seq results, revealing that the 3′ trimmed isomiRs dramatically increased with brain maturation (Figure 2C). Notably, our RT-qPCR pilot study (Supplementary Figure S1C) utilized primers targeting the canonical form of miR-338-3p, therefore we failed to detect the 3′ trimmed miR-338-3p isomiRs. This likely contributed to the discrepancy between our Combo-Seq and pilot study results.

We then applied our miR-338-3p isomiR-specific RT-qPCR assay to additional tissues from the same mice used in our Combo-Seq studies. We found that 3′ trimmed isomiRs increased with age in all the brain tissues tested but none of the other body tissues examined (Figures 2D,E), apart from a small increase in the −UG isomiR in kidney. This suggests that the increase in miR-338-3p trimming may be a specific feature of brain maturation.

In miRNA literature, 3′ trimming is often associated with miRNA degradation, suggesting that these miR-338-3p isomiRs might not be functional. During interactions with mRNA targets, inhibition is mediated by an RNA-protein complex known as the RISC, or RNA-induced silencing complex. AGO proteins are obligate components of this complex and directly bind to miRNAs during target interactions. We examined whether trimmed isomiRs associate with AGO proteins in the hippocampus by RNA co-immunoprecipitation (RIP) (Figure 2F). AGO-IP was confirmed by western blot and mass spectrometry (Figure 2G), and miR-338-3p isomiR RIP was measured by isomiR-specific RT-qPCR. All miR-338-3p isomiRs were enriched in the AGO-IP fraction relative to a bead only IP control (Figure 2H). Furthermore, miR-338-3p isomiRs were depleted in the corresponding flow-through sample, suggesting that a majority of each isomiR is bound to AGO proteins (Figure 2H). Finally, direct comparison of isomiRs revealed that the −G isomiR has the highest affinity for AGO, while the −UG isomiR has a slightly lower affinity than the other forms (Figure 2I). We conclude that all 3 isomiRs of miR-338-3p associate with AGO proteins in the mouse hippocampus.

Next, we used mimics to overexpress each miR-338-3p isomiR in Neuro-2a cells and examined their ability to inhibit miR-338-3p targets. RT-qPCR confirmed that mimics resulted in isomiR overexpression after transfection (Supplementary Figure S3A). Luciferase assays using the miR-338-3p FL mimic confirmed that the canonical form inhibits *Renilla* luciferase expression when the reporter mRNA contains a 3′UTR sequence from the miR-338-3p targets *Aatk* or *Drd2* (Kos et al., 2012; Chun et al., 2017), but not when the reporter lacks a target 3′UTR (Supplementary Figure S3B). Surprisingly, all isomiR mimics resulted in similar downregulation of the *Renilla* luciferase reporter with the *Drd2*-3′UTR (Supplementary Figure S3C). All isomiR mimics also produced similar downregulation of endogenous *Aatk* mRNA (Supplementary Figure S3D). Finally, isomiR mimics failed to affect *Nova1* mRNA, another known miR-338-3p target (Sun et al., 2021), but all resulted in NOVA1 protein downregulation (Supplementary Figures S3E–G). Together, these results suggest that the miR-338-3p FL, −G, and −UG isomiRs are functional in Neuro-2a cells, and we find no evidence that the 3′ trimming that produces the -G and -UG isomiRs interferes with miR-338-3p function.

### 2.3. MiRNA 3′ trimming increases and U tailing decreases during adolescent brain maturation

**Figure 3:**
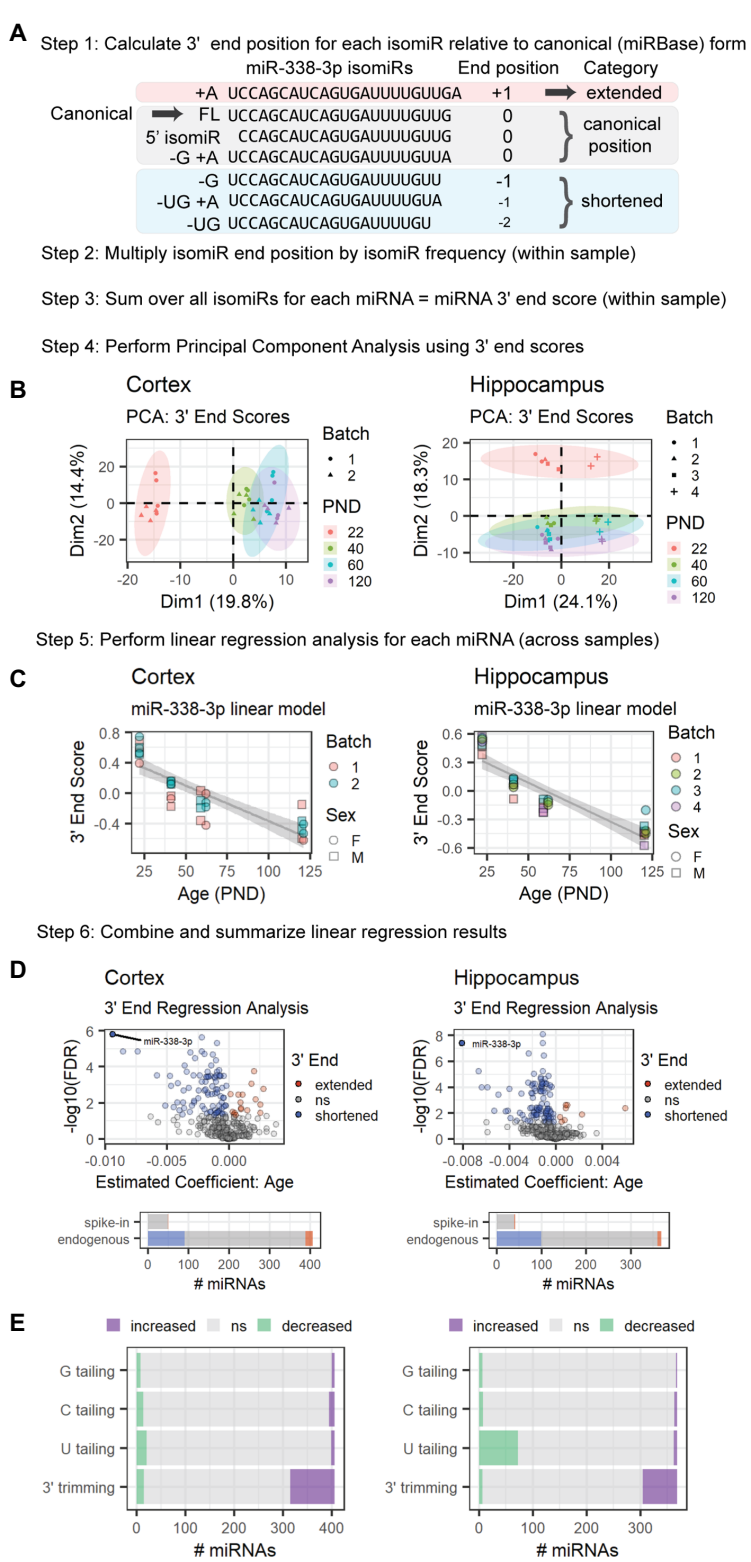
MiRNA 3′ ends undergo shortening between P22 and P120 in cortex and hippocampus due to increased 3′ trimming and decreased 3′ U tailing. This schematic provides an overview of the 3′ ERA method. MiR-338-3p is used as a representative example, but this method can be applied to any miRNA in a sequencing data set. **(A)** Alignment of miR-338-3p isomiRs relative to the canonical form, i.e., the sequence listed in miRBase. 5′ end variance is ignored in this analysis, as is sequence variance that does not affect miRNA length at the 3′ end. Negative 3′ end positions correspond with shortened 3′ ends; conversely, positive 3′ end positions correspond with extended 3′ ends. **(B)** PCA analysis is used to identify outlier samples and to determine whether age or other variables (e.g., batch effects or sex) strongly impact 3′ end scores. In the hippocampus data set, Batch 4 consistently differs from the other batches in Dim1, but samples are still clearly separated along Dim2 within each batch. No outliers were identified in either data set. **(C)** Significance is assessed for each miRNA *via* linear regression modeling: miRNA 3′ end score ~ Age + Batch + Sex. Variables will vary by experiment. Trend lines were fit using the geom_smooth() function in R, with method set to “lm.” A negative Estimate (Age) and negative slope in the trend line for miR-338-3p suggests that miR-338-3p undergoes 3′ shortening with age. **(D)** Volcano plots are used to summarize the linear regression modeling results for endogenous miRNAs. For both our cortical and hippocampal data sets, miR-338-3p undergoes the most 3′ shortening between P22 and P120. However, many miRNAs in both data sets undergo 3′ shortening (i.e., Estimate <0 and *p* < 0.05), whereas relatively few undergo 3′ extension (i.e., Estimate >0 and *p* < 0.05). Bar plots indicate the number of miRNAs in each category and include both endogenous and spike-in miRNAs. **(E)** Regression analysis may be applied to specific 3′ end modifications to identify the source of 3′ extension or shortening in endogenous miRNAs. In cortex (left), shortening is primarily the result of increased 3′ trimming, whereas shortening in hippocampus (right) is due to a combination of increased 3′ trimming and reduced U tailing. See Supplementary Figure S4 for sequencing position coverage demonstrating that miRNA 5′ end positions are stable with age whereas position coverage at the 3′ end is consistent with 3′ shortening in -3p and -5p miRNAs in both tissues.

Next, we examined whether the increase in miR-338-3p 3′ trimming was unique to miR-338-3p or part of a larger trend. To do so, we quantified age-associated changes in the length of the 3′ end of each miRNA using a method we will refer to as 3′End Regression Analysis (3′ERA) (Figure 3). We began by calculating the 3′ end position of each isomiR relative to the position of the 3′ terminal nucleotide of the miRNA’s canonical form (Figure 3A), defined as the sequence listed in miRBase for each miRNA (Griffiths-Jones et al., 2006, 2008; Kozomara and Griffiths-Jones, 2011, 2014; Kozomara et al., 2019). A negative position indicates that the 3′ end of an isomiR is shorter than the canonical form, whereas a positive position indicates that the isomiR is extended relative to the canonical form. Variance in the 5′ end of the miRNA was ignored, as were changes in nucleotide composition that do not affect 3′ length. A 3′ end score for each miRNA within each sample was then calculated by multiplying the 3′ end position by the frequency of the isomiR within each sample and summing over all isomiRs for that miRNA. Next, we performed PCA of the 3′ end scores to identify outlier samples and determine whether samples clustered by age. As shown in Figure 3B, the first dimension of the PCA of cortical samples and second dimension of the PCA of hippocampus samples were associated with the subject’s age. For the hippocampus samples, samples from the Batch 4 library preparation clustered separately from the other 3 library batches but displayed a similar pattern to the other batches in the second dimension.

Next, we used linear regression modeling to examine the relationship between age and each miRNA’s 3′ end score across samples. The results of this analysis for miR-338-3p are shown in Figure 3C. As predicted, the 3′ end score for miR-338-3p declines with age as the 3′ end of the miRNA shortens due to 3′ trimming. A similar analysis was performed for all miRNAs detected at a mean of 10 RPM across samples, and the results are compiled in Figure 3D. Notably, in both the cortex and hippocampus, miR-338-3p underwent the greatest 3′ end shortening during adolescent and early adult brain maturation. However, a large number of miRNAs in both data sets also significantly shortened with age, and relatively few 3′ ends were extended with age. None of the small spike-in RNAs exhibited age-associated shortening.

We also examined the overall position coverage in miRNA reads as a function of age. Consistent with an overall shortening of miRNA 3′ ends, the 3′ end position was shifted to the left in samples derived from older subjects relative to P22 (Supplementary Figures S4A,B). No shift was observed in reads derived from small spike-in RNAs. Also, no shift was observed in the 5′ end of miRNA reads, suggesting the position of the 5′ end was stable with age for most miRNAs (Supplementary Figures S4C,D). Finally, the 3′ end position is shifted to the left in both -3p and -5p miRNAs, suggesting 3′ end shortening occurs in miRNAs derived from both arms of pre-miRNAs (Supplementary Figures S4E,F). In the canonical miRNA synthesis pathway, the Microprocessor generates the 3′ ends of -3p miRNAs, while DICER generates the 3′ ends of -5p miRNAs (Bartel, 2018). Our observation that both −3p and −5p miRNAs undergo shortening with age suggests that shortening occurs in mature miRNAs.

MiRNA 3′ end variance is generated through multiple mechanisms, including 3′ trimming by 3′-5′ exonucleases and 3′ tailing by nucleotidyl transferases that typically add A or U nucleotides (Han and Mendell, 2022). MiR-338-3p shortening occurs due to an increase in 3′ trimming with age, but other miRNAs might undergo shortening due to a decrease in 3′ tailing with age. To distinguish between these mechanisms, we repeated 3′ERA but considered tailing and trimming separately. During Combo-Seq library preparation, poly(A) tails are added to miRNAs, and this poly(A) tail is used as a template during reverse transcription, similar to the RT-qPCR method shown in Figure 2A. As a result, 3′ terminal A nucleotides present prior to poly(A) tailing cannot be distinguished from those added during library preparation. Therefore, we did not perform regression analysis for A tailing. We found that the majority of 3′ shortening in the cortex was due to an increase in 3′ trimming with age (Figure 3E). In contrast, 3′ shortening in the hippocampus was due to a combination of increased 3′ trimming and decreased U tailing.

### 2.4. MiRNA 3′ shortening is associated with stronger correlations with target mRNA and protein levels

**Figure 4:**
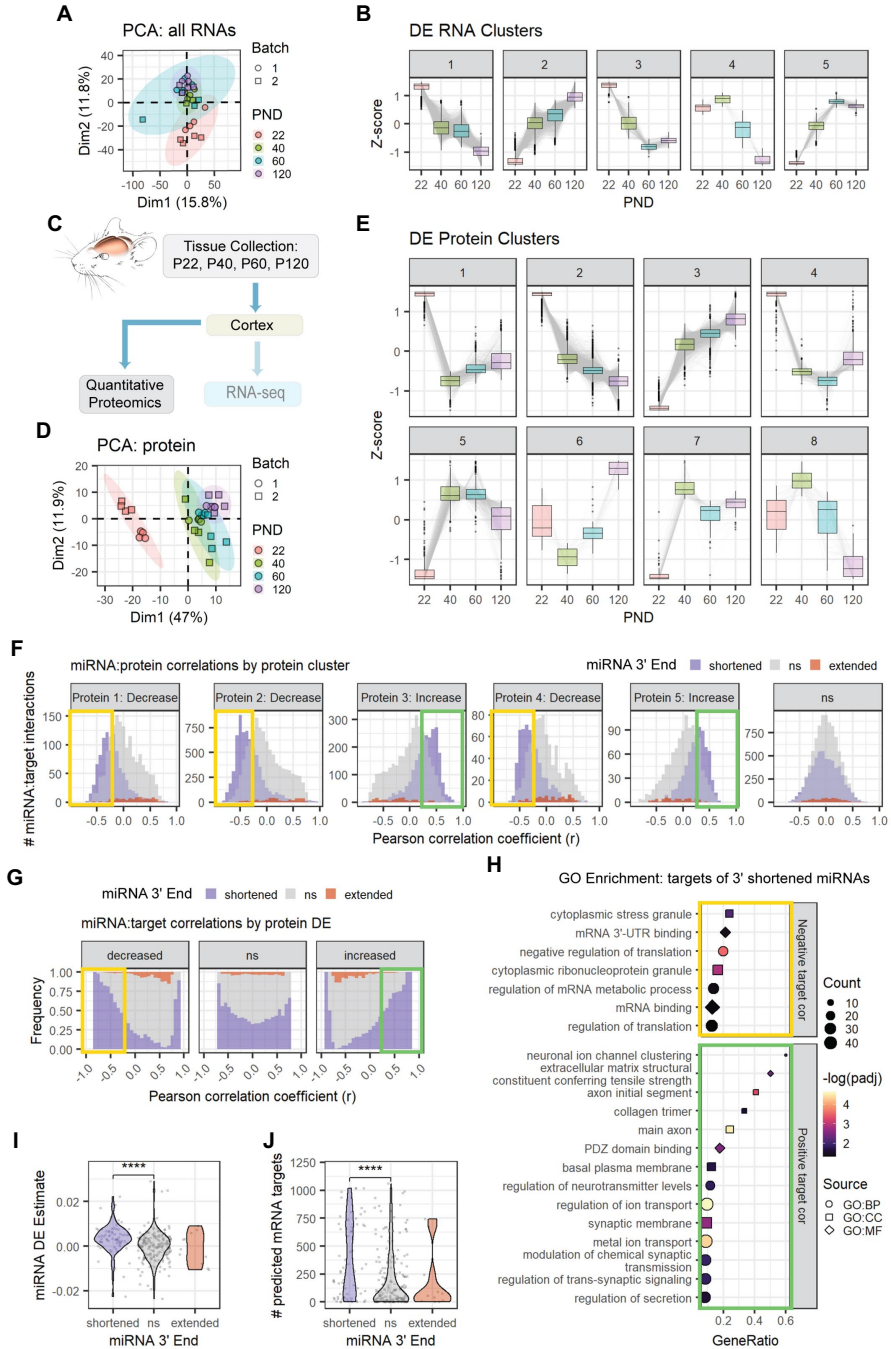
Age-associated 3′ shortening of miRNAs is associated with stronger correlations with target protein levels in cortex. **(A)** PCA was performed on count data for all RNAs in the cortex Combo-Seq data set, including mRNAs and miRNAs. QuagmiR-mapped counts were used for miRNAs. All other count data came from the exceRpt pipeline. **(B)** Clusters representing DE patterns for RNAs in cortex. **(C)** Cortical tissue sections were split after dissection, with half used for Combo-Seq and half used for quantitative proteomics. **(D)** PCA was performed on protein data in cortex proteomics data set. No outliers were identified. **(E)** Clusters representing DE patterns for proteins in cortex. **(F)** Pearson correlation analysis was performed for miRNAs and proteins encoded by their predicted targets in the cortex data set. Data are organized in histograms by protein cluster. *Y*-axis reflects the number of predicted miRNA:target interaction pairs at each Pearson correlation coefficient, r, value. Bar color reflects each miRNA’s 3′ end dynamics with age. **(G)** Pearson correlation analysis organized by protein DE results. *Y*-axis indicates the relative frequency of targeting by miRNAs that have shortened, extended, or stable (ns) 3′ ends with age. Bar color reflects miRNA 3′ end dynamics with age. **(H)** GO term enrichment analysis results for protein targets that decreased with age and exhibited negative correlations (*r* < −0.25, orange box in **F,G**) or increased with age and exhibited positive correlations (*r* > 0.25, green box in **F,G**) with miRNAs that underwent 3′ shortening. Shapes indicate term sub-ontology. BP, biological process. CC, cellular component. MF, molecular function. **(I)** Violin plot representing the relationship between miRNA 3′ end dynamics with age and the miRNA’s DE Estimate, which summarizes the change in miRNA levels with age. Positive DE Estimate values reflect miRNAs that increase with age, while negative values reflect miRNAs that decrease with age. Each point represents one miRNA. Kruskal-Wallis: *p* = 8.7e-6. Wilcox test for pairwise comparisons: ****p < 0.0001. **(J)** Violin plot representing the relationship between miRNA 3′ end dynamics with age and the number of predicted mRNA targets detected for each miRNA. Each point represents one miRNA. Kruskal-Wallis: *p* = 0.00015. Wilcox test for pairwise comparisons: ****p < 0.0001. See Supplementary Figures S5, S6 for additional analyses of the cortex Combo-Seq and proteomics data sets. See Supplementary Figure S7 for corresponding hippocampus Combo-Seq data. See Supplementary Figure S8 for additional correlation analyses for the cortex data sets.

Next, we examined the relationship between miRNA 3′ shortening and miRNA targeting. To do so, we examined the count data in our Combo-Seq data. PCA plots demonstrate the samples cluster by age (Cortex: Figure 4A, Hippocampus: Supplementary Figure S7A). We then performed DE analysis followed by clustering analysis on RNAs that were differentially expressed with age (Cortex: Figure 4B, Hippocampus: Supplementary Figure S7B). The majority of these RNAs exhibited approximately linear decreases or increases with age, though some exhibited more complex patterns. mRNAs made up the majority of RNAs in each cluster. Over 200 small nucleolar RNAs (snoRNAs) were identified in Cluster 2 (Cortex: Supplementary Figure S5A, Hippocampus: Supplementary Figure S7C), suggesting a significant increase in overall snoRNA levels during adolescent and early adult brain maturation. snoRNAs function in rRNA biogenesis and direct modification of pre-rRNAs, pre-mRNAs, and snRNAs (Kufel and Grzechnik, 2019). In the cortex, Gene Ontology (GO) enrichment analysis also identified enrichment of mRNAs associated with mRNA translation in Cluster 2 and mRNAs encoding ribosomal proteins in Cluster 5 (Supplementary Figure S5C). These results suggest that a widespread upregulation of multiple RNA families critical for protein synthesis occurs during adolescent brain development. Notably, Cluster 2 also contained the highest number of miRNAs that undergo 3′ shortening with age, suggesting a relationship between miRNA 3′ end shortening and DE with age (Cortex: Supplementary Figure S5B, Hippocampus: Supplementary Figure S7D). By contrast, GO enrichment analysis of Cluster 1 suggests a downregulation of mRNAs associated with neurodevelopment (e.g., “oligodendrocyte differentiation” and “negative regulation of neurogenesis”) and extracellular matrix (ECM) regulation (Supplementary Figure S5C).

Following tissue collection, the cortical samples collected for Combo-Seq were split, and half of the sample was used for quantitative proteomics by tandem mass tag mass spectrometry (TMT-MS) (Figure 4C). PCA revealed that the samples clustered by age (Figure 4D). We then performed DE analysis followed by clustering analysis on proteins that were differentially expressed with age (Figure 4E). The largest clusters contained proteins that showed simple decreases (Cluster 2) or increases (Cluster 3) across all time points. GO enrichment analysis revealed that Cluster 2 was enriched for proteins associated with cytosolic and mitochondrial mRNA translation, mRNA splicing, and RNA binding (Supplementary Figure S6A). Most components of the canonical miRNA biogenesis pathway also decreased with age (Supplementary Figure S6B). The decreases in DGCR8 and DICER were validated by western blot (Supplementary Figure S6C). Cluster 3 was enriched with proteins associated with myelination, synaptic activity, cellular respiration, and ribonucleotide synthesis. Enrichment of synaptic proteins was also observed in Cluster 5, which contains proteins that increased between P22 and P40, i.e., early adolescence.

Together, these results suggest a major shift in the pathways that regulate the synthesis, function, and stability of RNAs. A surprising number of mRNAs showed divergent expression patterns with their encoded proteins. For example, many of the mRNAs that encode ribosomal proteins increased with age, but ribosomal proteins declined with age. We calculated Pearson correlation coefficients for all mRNA:protein pairs in our cortical data and observed that mRNA DE poorly predicted protein DE (Supplementary Figure S8A). Although mRNAs that decreased with age often showed modest positive correlations with their encoded proteins, mRNAs that increased with age were equally likely to show positive or negative correlations with their encoded proteins.

We then examined the relationship between miRNAs, the mRNAs they target, and the proteins those targets encode (i.e., “protein targets”). Overall, miRNA levels were poorly correlated with their mRNA targets (Cortex: Supplementary Figure S8B, Hippocampus: Supplementary Figure S7E). However, miRNA levels showed stronger correlations with the proteins encoded by their mRNA targets (Supplementary Figure S8C). As expected, miRNAs that increased with age (RNA Clusters 2 and 5) exhibited moderate negative correlations with protein targets. By contrast, miRNAs that decreased with age exhibited more positive correlations with protein targets, i.e., the proteins also decreased with age.

When we examined the relationship between miRNA 3′ end dynamics and target correlations, a surprising pattern emerged (Cortex mRNA: Supplementary Figures S8D,E, Cortex protein: Figures 4F,G, Hippocampus mRNA: Supplementary Figures S7F,G). MiRNAs that underwent 3′ shortening with age exhibited stronger negative correlations with targets that decreased with age and stronger positive correlations with targets that increased with age than miRNAs that had stable 3′ ends or miRNAs that underwent 3′ extension with age. This pattern was apparent when examining either mRNA targets or proteins encoded by mRNA targets. GO enrichment analysis of these negatively correlated protein targets in the cortex revealed enrichment of proteins involved in stress granules, mRNA translation, and mRNA-3′UTR binding (Figure 4H), including RISC-components TNRC6B and TNRC6C. The negatively correlated mRNA targets were associated with the ECM in the cortex (Supplementary Figure S8F) and with neurodevelopment in the hippocampus (Supplementary Figure S7H). In the cortex, upregulated, positively correlated target proteins of miRNAs that underwent 3′ shortening were associated with synaptic transmission and ion channel activity (Figure 4H), while mRNAs were associated with chromatin binding and the development of neuron projections (Supplementary Figure S8F). Notably, miRNA 3′ end dynamics showed no relationship with target correlations when we examined mRNA or protein targets that were not DE with age.

MiRNAs that increase with age have negative correlations with targets that decrease with age and positive correlations with targets that increase with age. MiRNAs that underwent 3′ shortening with age were most abundant in RNA Cluster 2, i.e., RNAs that increased with age across adolescent and early adult brain maturation (Cortex: Supplementary Figure S5A, Hippocampus: Supplementary Figure S7D). Overall, miRNAs that underwent 3′ shortening with age were more likely to increase with age than were miRNAs with stable 3′ ends (Cortex: Figure 4I, Hippocampus: Supplementary Figure S7I). Furthermore, miRNAs that underwent 3′ shortening with age also had significantly more predicted mRNA targets expressed in our data sets (Cortex: Figure 4J, Hippocampus: Supplementary Figure S7J). Both findings might contribute to our observation that miRNAs which underwent 3′ shortening exhibited stronger correlations with DE targets.

### 2.5. MiRNA 3′ shortening increases in mouse cortex between P10 and P28, but not between P4 and P14

**Figure 5:**
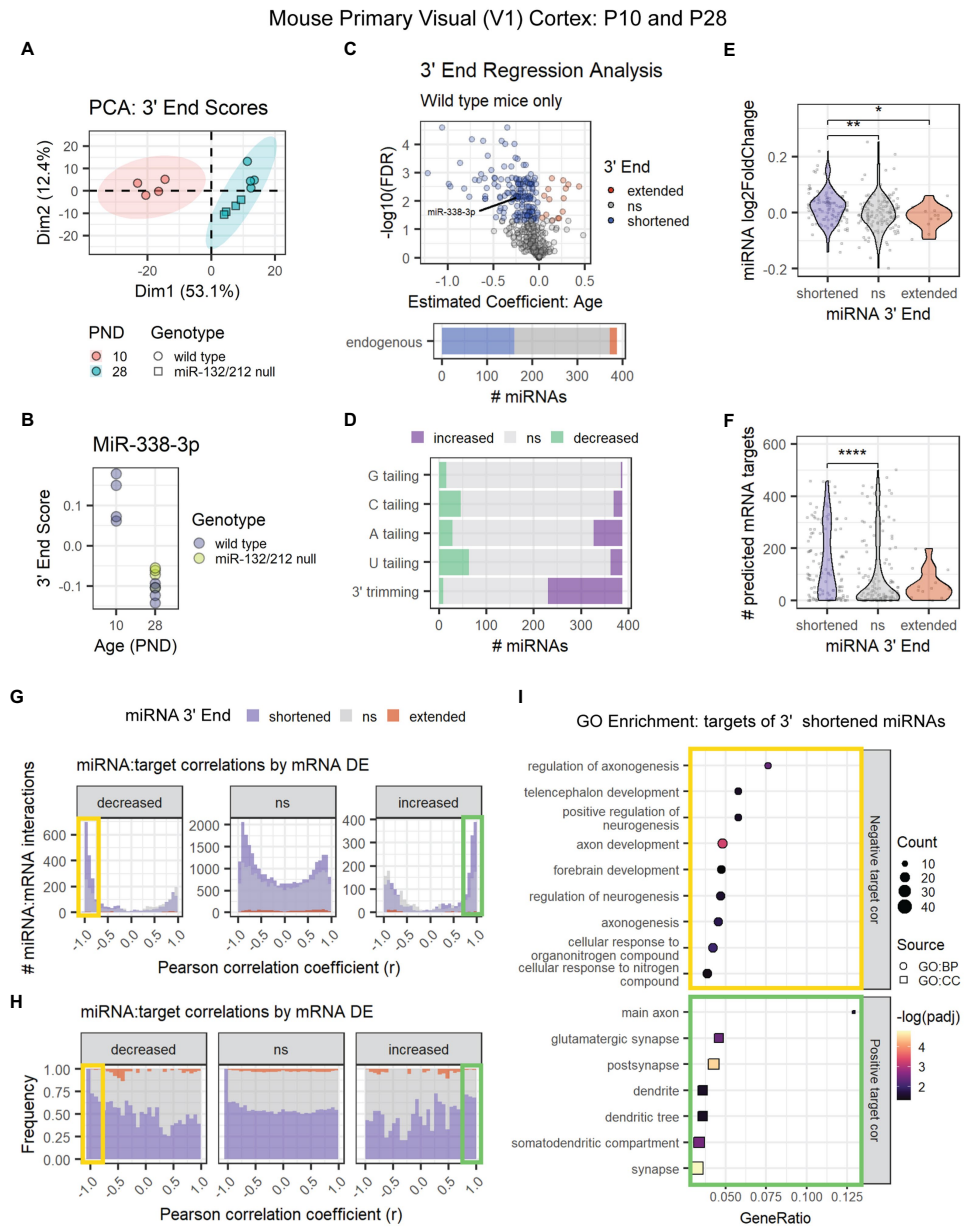
Age-associated 3′ shortening of miRNAs occurs in mouse V1 cortex between P10 and P28. **(A)** PCA was performed on 3′ end scores derived from miRNA-seq data from P10 and P28 mouse V1 cortex. Samples clustered by age. No outliers were identified. **(B)** MiR-338-3p 3′ end scores decrease between P10 and P28. **(C)** 3′ERA results demonstrate that ~40% of miRNAs exhibit increased 3′ shortening. **(D)** 3′ERA results were subset by 3′ modification type. 3′ shortening is primarily the result of increased 3′ trimming with age. **(E)** Violin plot representing the relationship between miRNA 3′ end dynamics with age and the miRNA’s log_2_ fold change between P10 and P28. Each point represents one miRNA. Kruskal-Wallis: *p* = 0.0058. Wilcox test for pairwise comparisons: *p < 0.05, **p < 0.01. **(F)** Violin plot representing the relationship between miRNA 3′ end dynamics with age and the number of predicted targets detected in mRNA-seq data. Each point represents one miRNA. Kruskal-Wallis: *p* = 0.00018. Wilcox test for pairwise comparisons: ****p < 0.0001. **(G)** Pearson correlation analysis was performed for miRNAs and their predicted mRNA targets. Data are organized in histograms by mRNA DE cluster. *Y*-axis reflects the number of predicted miRNA:target interaction pairs at each Pearson correlation coefficient, r, value. Bar color reflects each miRNA’s 3′ end dynamics with age. **(H)** Pearson correlation analysis organized by mRNA DE results. *Y*-axis indicates the relative frequency of targeting by miRNAs that have shortened, extended, or stable (ns) 3′ ends with age. Bar color reflects miRNA 3′ end dynamics with age. **(I)** GO term enrichment analysis results for mRNA targets that decreased with age and exhibited negative correlations (*r* < −0.75, orange box in **G,H**) or increased with age and exhibited positive correlations (*r* > 0.75, green box in **G,H**) with miRNAs that underwent 3′ shortening.

MiRNA 3′ shortening might be a unique feature of adolescent and early adult brain maturation or a general pattern in mammalian brain aging. To determine whether 3′ shortening also occurs prior to adolescence, we conducted 3′ERA on a previously published miRNA-seq data set from mouse primary visual (V1) cortex at P10 and P28 (Mazziotti et al., 2017). PCA revealed that the samples clustered by the subject’s age (Figure 5A). As in adolescence, miR-338-3p 3′ end scores decreased between P10 and P28 (Figure 5B), suggesting that the miR-338-3p 3′ trimming begins prior to adolescence. Furthermore, ~40% of miRNAs exhibited significant 3′ shortening in this data set, primarily due to an increase in 3′ trimming with age (Figures 5C,D).

To determine whether 3′ end dynamics also influences target correlations, we analyzed the associated miRNA and mRNA count data from the V1 cortex of P10 and P28 wild type mice. MiRNAs that exhibited 3′ shortening with age were more likely to increase with age than miRNAs with stable or extended 3′ ends (Figure 5E) and contained significantly more predicted mRNA targets in the mRNA data set (Figure 5F). We then calculated Pearson correlations for each miRNA and its predicted mRNA targets. As observed in adolescent brain maturation, we found that miRNAs that underwent 3′ shortening exhibited stronger negative correlations with mRNA targets that decreased with age and stronger positive correlations with mRNA targets that increased with age (Figures 5G,H). GO enrichment analysis revealed that downregulated, negatively correlated targets of miRNAs that underwent 3′ shortening were associated with neurogenesis and neurite outgrowth (Figure 5I). Upregulated, positively correlated targets were associated with cellular components of synapses, dendrites, and axons.

**Figure 6:**
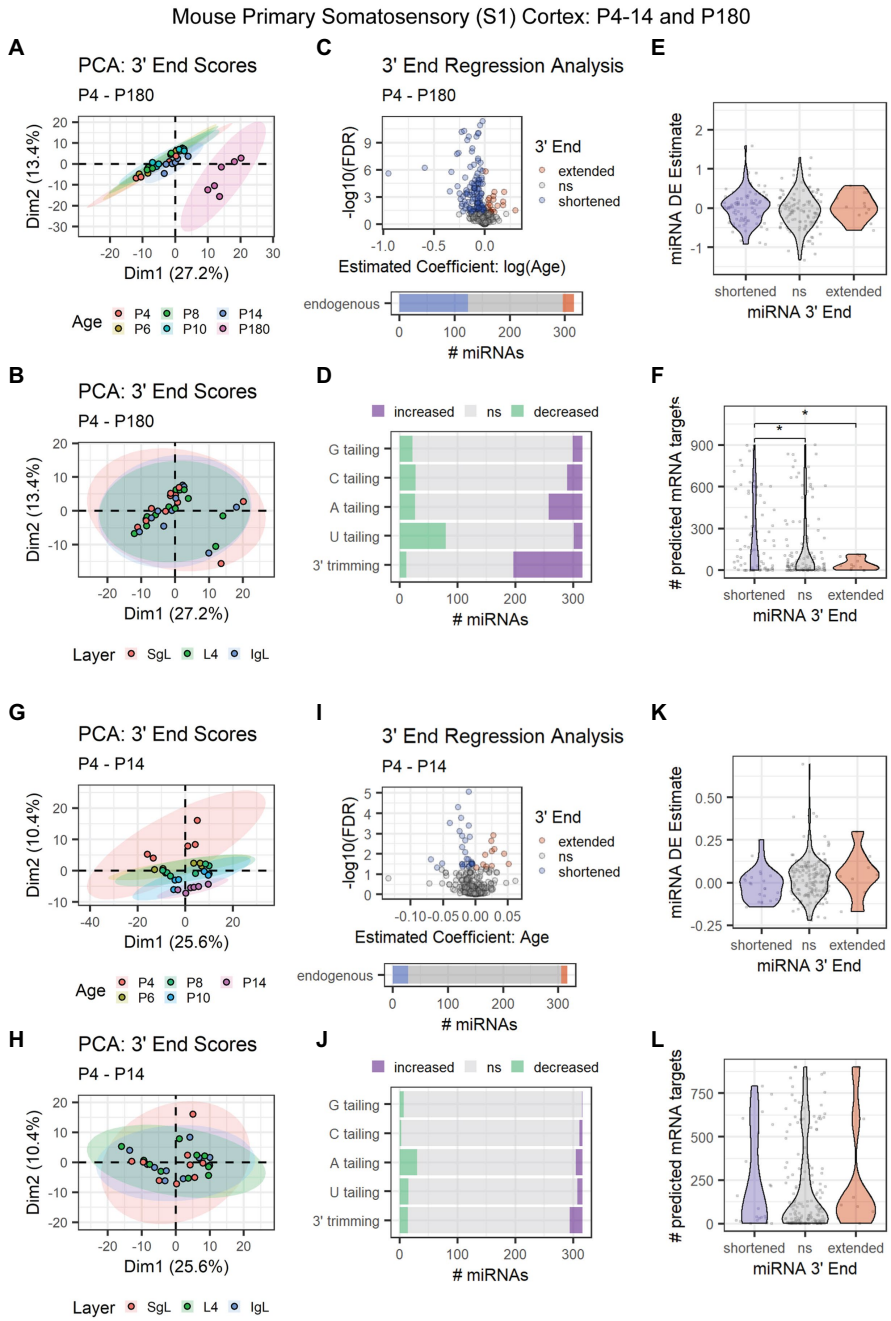
Age-associated 3′ shortening of miRNAs occurs in mouse S1 cortex between P14 and P180, but not between P4 and P14. **(A–F)** Data derived from P4, P6, P8, P10, P14, and P180 samples were included. For G-L, P180 samples were excluded. **(A)** PCA was performed on 3′ end scores derived from miRNA-seq data from P4-14 and P180 mouse S1 cortex. Samples clustered by age, with P180 samples clustering apart from earlier time points. Sample SRR1016226 was identified as an outlier and removed from downstream analyses (sample not shown). **(B)** PCA on 3′ end scores from P4–180 mice revealed no relationship with cortical layer in the first two dimensions. **(C)** 3′ERA for mouse S1 cortex between P4 and P180. 124 of 316 miRNAs underwent 3′ shortening with age. **(D)** 3′ERA by modification type revealed that 3′ shortening between P4 and P180 was primarily due to increased 3′ trimming and decreased U tailing. **(E)** Violin plot representing the relationship between miRNA 3′ end dynamics between P4 and P180 and the miRNA’s DE Estimate, which summarizes the change in miRNA levels with age. Positive DE Estimate values reflect miRNAs that increase with age, while negative values reflect miRNAs that decrease with age. Each point represents one miRNA. Kruskal-Wallis: *p* = 0.5. **(F)** Violin plot representing the relationship between miRNA 3′ end dynamics between P4 and P180 and the number of predicted mRNA targets detected for each miRNA. Each point represents one miRNA. Kruskal-Wallis: *p* = 0.011. Wilcox test for pairwise comparisons: *p < 0.01. **(G)** PCA was performed on 3′ end scores derived from miRNA-seq data from P4-14 mouse S1 cortex. Samples approximately clustered by age. **(H)** PCA on 3′ end scores from P4-14 mice revealed no relationship with cortical layer in the first two dimensions. **(I)** 3′ERA for mouse S1 cortex between P4 and P14. Only 28 of 316 miRNAs underwent 3′ shortening with age. **(J)** 3′ERA by modification type revealed that increased A tailing was the single largest contributor to 3′ end dynamics between P4 and P14. **(K)** Violin plot representing the relationship between miRNA 3′ end dynamics between P4 and P14 and the miRNA’s DE Estimate, which summarizes the change in miRNA levels with age. Positive DE Estimate values reflect miRNAs that increase with age, while negative values reflect miRNAs that decrease with age. Each point represents one miRNA. Kruskal-Wallis: *p* = 0.13. **(L)** Violin plot representing the relationship between miRNA 3′ end dynamics between P4 and P14 and the number of predicted mRNA targets detected for each miRNA. Each point represents one miRNA. Kruskal-Wallis: *p* = 0.51. See Supplementary Figure S9 for analysis of P4-14 miRNA and mRNA count data and target correlations.

To pinpoint the onset of 3′ shortening in mouse brain development, we conducted 3′ERA on miRNA-seq data (SRA: SRP031888) derived from P4, P6, P8, P10, P14, and P180 mouse primary somatosensory (S1) cortex (Fertuzinhos et al., 2014). Adult-derived samples clearly segregated from the earlier time points in a PCA of 3′ end scores (Figure 6A). No relationship between 3′ end scores and the cortical layer was observed in the first two dimensions of our PCA (Figure 6B). Over 40% of miRNAs underwent 3′ shortening with age due to a combination of increased 3′ trimming and decreased U tailing (Figures 6C,D). Although 3′ end dynamics did not predict miRNA DE (Figure 6E), miRNAs that underwent 3′ shortening had significantly more predicted targets expressed in the corresponding mRNA-seq data set from these subjects (Figure 6F).

Based on PCA results, we hypothesized that the increase in 3′ shortening observed between P4 and P180 was primarily driven by brain maturation after P14. We, therefore, repeated our analysis but excluded adult time points to focus on changes between P4 and P14, prior to the onset of adolescence. Samples approximately clustered by subject age in a PCA, but ages substantially overlapped (Figure 6G). We found no relationship between cortical layers and 3′ end score PCA results (Figure 6H). Fewer than 10% of miRNAs exhibited 3′ shortening between P4 and P14, consistent with our hypothesis that shortening primarily occurred between P14 and P180 in our previous analysis (Figure 6I). Similarly, relatively few miRNAs exhibited increased 3′ trimming or decreased U tailing. Decreased A tailing appeared to be the biggest contributor to the shortening that was observed (Figure 6J). No relationship was observed between 3′ end dynamics and miRNA DE (Figure 6K) or the number of expressed mRNA targets (Figure 6L).

We also examined count data for miRNAs and mRNAs in S1 cortex between P4 and P14. Samples clustered by both age and cortical layer (dimensions 1 and 2, respectively) (Supplementary Figures S9A,B). Clustering analysis revealed complex patterns in both miRNA and mRNA expression during these time points (Supplementary Figures S9C,D). However, miRNAs and their mRNA targets were poorly correlated, regardless of miRNA 3′ end dynamics (Supplementary Figures S9E,F).

Together, these results suggest that miRNA 3′ shortening begins between P14 and P28 in the mouse cortex and continues into adulthood.

### 2.6. Age-associated miRNA 3′ shortening occurs in human cortex

Finally, we examined whether 3′ shortening of miRNAs also occurs during human development and aging. We identified two miRNA-seq data sets derived from human superior frontal gyrus for 3′ERA.

**Figure 7:**
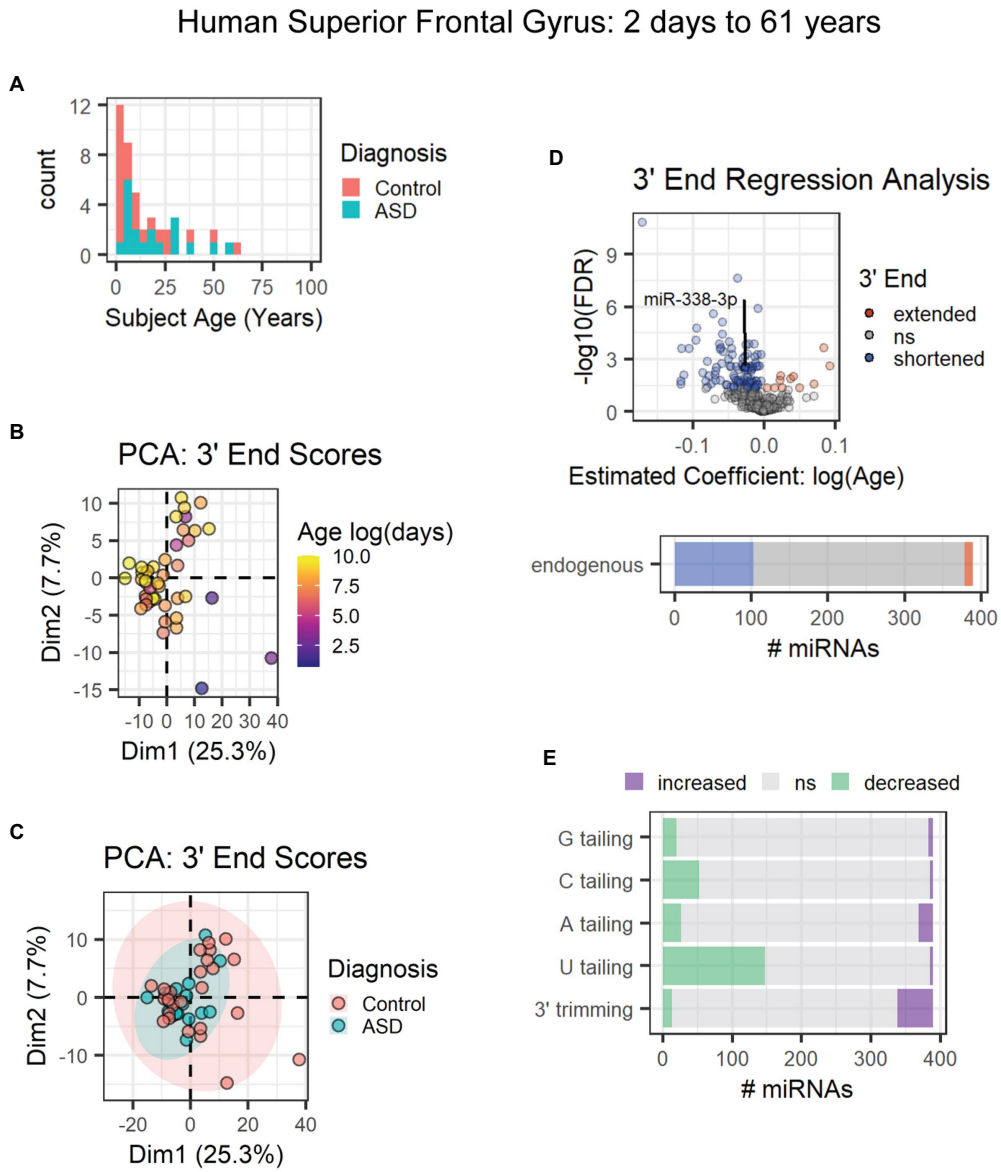
Age-associated 3′ shortening of miRNAs occurs in human superior frontal gyrus between 2 days and 61 years of age. **(A)** Age distribution and diagnosis information for human subjects used for analyses in **(B–E)**. After outlier removal, 44 subjects were included. **(B)** PCA suggests a weak relationship between subject age and 3′ end scores. Sample SRR1510086 was removed as an outlier based on PCA results (not shown). **(C)** PCA suggests no relationship between subject diagnosis and 3′ end scores in the first two dimensions. **(D)** 3′ERA for human cortex. Ages were log transformed prior to analysis. 103 of 389 miRNAs underwent 3′ shortening with age. **(E)** 3′ERA by modification type revealed that 3′ shortening was primarily due to increased 3′ trimming and decreased U tailing with age.

The first data set included subjects ranging from 2 days to 61 years of age from a combination of neurotypical subjects and those diagnosed with autism spectrum disorder (Figure 7A) [GEO data set: GSE59286 (unpublished)]. PCA results suggested that age and not diagnosis primarily influenced 3′ end scores (Figures 7B,C). However, diagnosis was included as a covariate in our regression analysis. Over 100 miRNAs (~25%), including miR-338-3p, exhibited 3′ shortening with age, and few exhibited 3′ extension with age (Figure 7D). 3′ shortening was due to a combination of increased 3′ trimming and decreased U tailing with age (Figure 7E). However, over twice as many miRNAs exhibited reduced U tailing than increased trimming. No mRNA target data were available for these subjects. Hence, correlation analysis was not performed.

**Figure 8:**
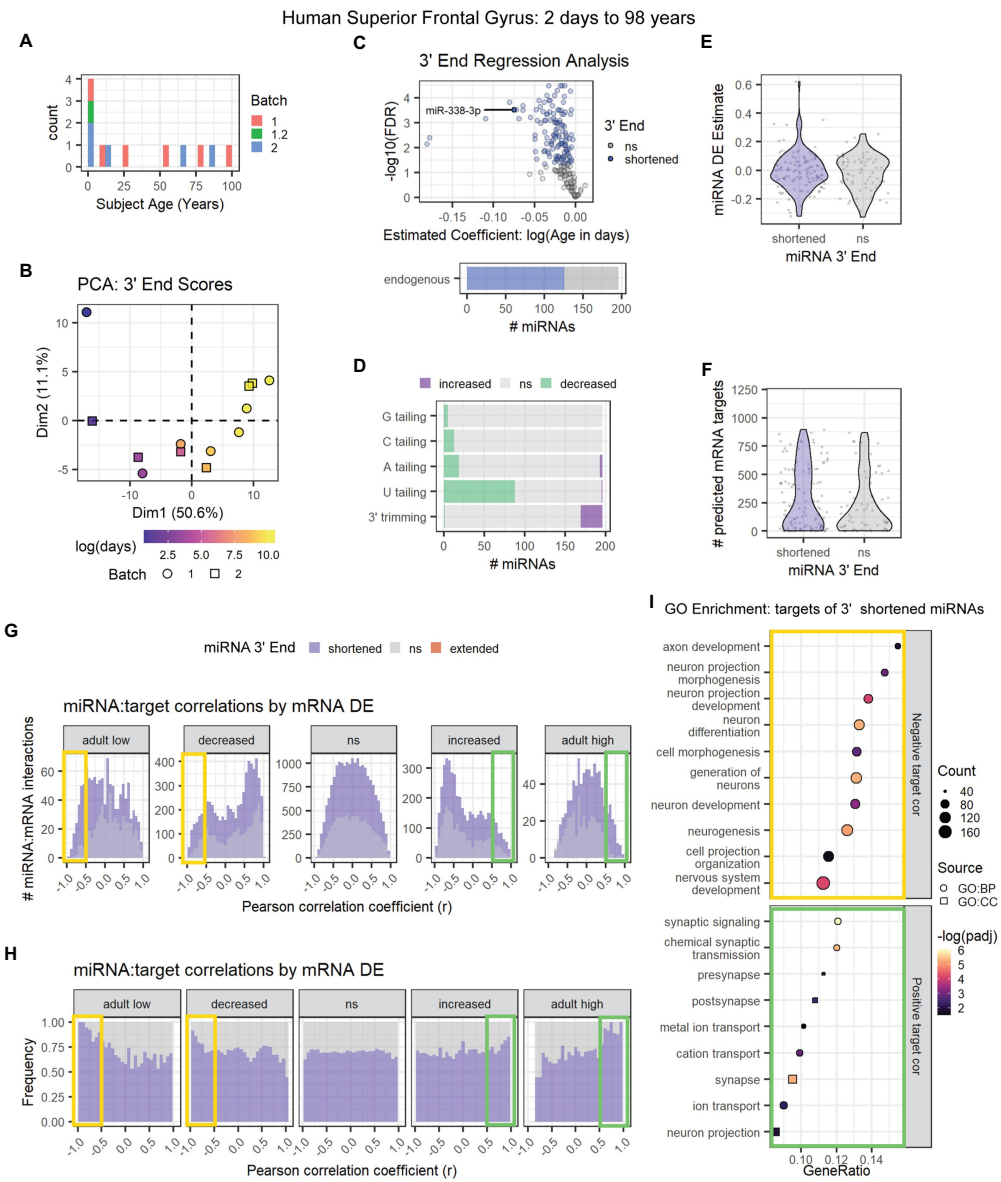
Age-associated 3′ shortening of miRNAs occurs in human superior frontal gyrus between 2 days and 98 years of age. **(A)** Age distribution for human subjects used for analyses in A-I. After outlier removal, 12 subjects were included. **(B)** Samples approximately align by subject age in a PCA plot of 3′ end scores. Sample GSM450608 was removed as an outlier based on PCA results (not shown). **(C)** 3′ERA for human cortex. 126 of 196 miRNAs underwent 3′ shortening with age. **(D)** 3′ERA by modification type revealed that 3′ shortening was primarily due to increased 3′ trimming and decreased U tailing with age. **(E)** Violin plot representing the relationship between miRNA 3′ end dynamics and the miRNA’s DE Estimate, which summarizes the change in miRNA levels with age. Positive DE Estimate values reflect miRNAs that increase with age, while negative values reflect miRNAs that decrease with age. Each point represents one miRNA. Wilcox test: *p* = 0.8. **(F)** Violin plot representing the relationship between miRNA 3′ end dynamics and the number of predicted mRNA targets detected for each miRNA. Each point represents one miRNA. Wilcox test: *p* = 0.17. **(G)** Pearson correlation analysis was performed for miRNAs and their predicted mRNA targets. Data are organized in histograms by mRNA DE results (See Supplementary Figure S10D for corresponding cluster expression patterns). *Y*-axis reflects the number of predicted miRNA:target interaction pairs at each Pearson correlation coefficient, r, value. Bar color reflects each miRNA’s 3′ end dynamics with age. **(H)** Pearson correlation analysis organized by mRNA DE results. *Y*-axis indicates the relative frequency of targeting by miRNAs that have shortened or stable (ns) 3′ ends with age. No miRNAs exhibited 3′ extension with age. Bar color reflects miRNA 3′ end dynamics with age. **(I)** GO term enrichment analysis results for mRNA targets that decreased with age and exhibited negative correlations (*r* < −0.5, orange box in **G,H**) or increased with age and exhibited positive correlations (*r* > 0.5, green box in **G,H**) with miRNAs that underwent 3′ shortening. See Supplementary Figure S10 for PCA results and cluster expression patterns for miRNA-seq and mRNA microarray expression data used in the targeting analysis.

The second human data set (GSE18012) included neurotypical subjects ranging from 2 days to 98 years of age (Figure 8A; Somel et al., 2010). PCA results were consistent with an association between age and miRNA 3′ end dynamics (Figure 8B). Almost 60% of miRNAs exhibited significant 3′ shortening with age including miR-338-3p (Figure 8C), due to a combination of decreased U tailing and increased 3′ trimming (Figure 8D). As with the first human data set, decreased U tailing appeared to be the primary source of 3′ shortening. 3′ end dynamics were not associated with miRNA DE or with the number of mRNA targets detected in mRNA microarray data from the same subjects (Figures 8E,F).

We then examined the relationship between 3′ end dynamics and miRNA:target correlations. Expression data for miRNAs and mRNAs revealed a high number of DE clustering patterns (Supplementary Figures S10A–D). mRNA clusters with similar expression patterns were combined for the analysis shown in Figures 8G,H (See Supplementary Figure S10E for correlation results by mRNA cluster). As observed in previous data sets, miRNAs that underwent 3′ shortening with age exhibited stronger negative correlations with mRNAs that decreased with age and stronger positive correlations with mRNAs that increased with age, than miRNAs that did not undergo 3′ shortening with age.

GO enrichment analysis (Figure 8I) revealed that downregulated, negatively correlated mRNA targets of miRNAs that underwent 3′ shortening with age were associated with neuronal development, e.g., neurogenesis, neuronal differentiation, and neurite outgrowth. Upregulated, positively correlated targets were associated with synaptic transmission and ion channel activity.

Based on these two data sets, we conclude that miRNA 3′ shortening increases with age in the human cortex and is associated with stronger correlations with mRNA target levels.

## 3. Discussion

Using combined mRNA/miRNA sequencing and quantitative proteomics, we examined miRNAs and their targets across multiple brain regions during adolescence and early adulthood in mice. We found that miRNA 3′ ends are shortened with age due to a combination of increased 3′ trimming and decreased U tailing. MiR-338-3p, a miRNA previously associated with thalamocortical deficits in a mouse model of 22q11DS (Chun et al., 2017), underwent the most 3′ trimming of all miRNAs examined in the mouse cortex and hippocampus at this time. Molecular assays confirmed that 3′ end trimming of 1 or 2 nucleotides does not impede miR-338-3p function. Furthermore, miRNAs that underwent 3′ shortening with age exhibited stronger correlations with mRNA targets, thus suggesting a relationship between 3′ end dynamics and target interactions during brain maturation. Finally, our data mining experiments demonstrated that age-associated miRNA 3′ shortening is conserved between mice and humans, is highly replicable across sequencing data sets, and occurs during multiple stages of mammalian postnatal brain development.

Our pilot studies should serve as a valuable cautionary tale for researchers examining miRNAs during neurodevelopment and aging. Both RT-qPCR and microarray data suggested that miR-338-3p underwent a modest decrease during adolescence and early adulthood. In contrast, sequencing results revealed that total miR-338-3p levels increased over 3-fold. This discrepancy appears to be due to the increase in the −G and −UG 3′ trimmed isomiRs of miR-338-3p, which were not detected using a standard RT-qPCR assay targeting the miR-338-3p canonical sequence. Detection of trimmed isomiRs was also impaired using a locked nucleic acid-based assay (Figure 2B). It is unclear why the miRNA microarray also failed to detect the increase in miR-338-3p with age. Microarray probes are also designed to target the miRBase-listed canonical miRNA sequence. Therefore, 3′ trimming may have also interfered with miR-338-3p detection by microarray, but further studies are needed to confirm this. When we modified our RT-qPCR assay to detect miR-338-3p isomiRs, our RT-qPCR results closely mirrored those obtained by miRNA sequencing. We therefore conclude that total miR-338-3p levels increase during adolescence and early adult brain maturation.

Our pilot study also suggested that overall miRNA levels declined with age; however, our Combo-Seq results demonstrated that overall miRNA levels increased with age relative to spike-in RNAs. This increase appears to be driven by a subset of highly abundant miRNAs rather than a global increase that affects most miRNAs. Several previous studies have attempted to examine changes in overall miRNA levels between early postnatal development and adulthood in humans or rodent models, but their results have been contradictory (Thomas and Zakharenko, 2021), perhaps due to differences in detection methods, data normalization, and the presence or absence of spike-in controls. Few of these studies have included adolescent subjects. Therefore, changes in miRNA levels during adolescence were largely inferred based on measurements from early postnatal subjects (or children) and adults. Our data suggest that overall miRNA levels and levels of individual miRNAs are highly dynamic across multiple stages of adolescence in the mouse cortex and hippocampus. Further research is needed to elucidate miRNA dynamics during human adolescence.

Our data suggest a fundamental shift in RNA pathways with age. Although overall miRNA levels increased, our quantitative proteomics data demonstrated that most of the proteins involved in the miRNA biogenesis pathway decreased with age, while the lincRNA *Neat1*, which enhances Microprocessor activity (Jiang et al., 2017), increased with age. Our Combo-Seq results also demonstrated that snoRNAs increased with age. SnoRNAs function in rRNA biogenesis and in guiding the modification of rRNAs, tRNAs, snRNAs, and mRNAs (Kufel and Grzechnik, 2019). Proteins involved in RNA nucleotide synthesis increased with age. A small subset of cytoplasmic and mitochondrial tRNAs also increased with age. While the mRNAs that encode ribosomal proteins increased with age, the proteins themselves decreased, as did other RNA binding proteins that regulate mRNA splicing and mRNA translation. Previous studies on mammalian brain development have found that protein synthesis peaks during early postnatal development and declines during adolescence and early adulthood (Fando et al., 1980; Vargas and Castañeda, 1983; Sun et al., 1995; Qin et al., 2005; Hovda et al., 2006). We speculate that an increase in miRNA levels and decrease in ribosomal proteins during adolescence might contribute to this decline in protein synthesis rates. Why other RNAs that regulate RNA biogenesis and protein synthesis increase with age remains unclear; however, this suggests that the relative importance of post-transcriptional regulation of gene expression may increase during adolescent and early adult brain maturation.

Our most robust finding was that miRNA 3′ ends shortened with age. Although many previous studies have examined changes in miRNA levels with age, very few have examined isomiR variation. A single study in mice observed that miRNA length declines between embryonic development (E12.5) and the onset of adulthood (P60) due to shortening at the 3′ end (Juvvuna et al., 2012). During adolescence and early adulthood, we observed that miRNA shortening occurred in the cortex primarily due to increased 3′ trimming and in the hippocampus due to a combination of increased 3′ trimming and decreased U tailing. Data mining of additional mouse miRNA sequencing data sets demonstrated that 3′ ends were stable in the mouse S1 cortex between P4 and P14 but shortened in the V1 cortex between P10 and P28 due to increased 3′ trimming. This suggests that miRNA 3′ shortening begins between P14 and P28, i.e., pre- or early adolescence, and extends into adulthood in mouse brain. We also observed miRNA 3′ shortening with age in two human data sets, but these data sets did not contain sufficient subjects to determine when in human development this shortening begins or ends.

The mechanism underlying miRNA 3′ shortening during brain maturation remains unknown. Juvvuna et al. (2012) attributed 3′ shortening to a change in the relative levels of AGO2 and AGO1, but AGO1-4 levels were stable with age in our proteomics data (Supplementary Figure S6B). We observed that several TUT proteins, which mediate miRNA U tailing, decrease with age in cortex, but we only observed a decrease in U tailing in the hippocampus and not in cortex. No known 3′-5′ exoribonucleases that target miRNAs increased with age in the cortex. Hence, it is unclear why trimming increased with age.

The functional consequences of miRNA 3′ shortening also remain unclear. Although miRNA trimming is often synonymous with miRNA degradation, our data demonstrate that miR-338-3p isomiRs are functional. Our AGO-RIP experiments suggest that 3′ trimming has a small effect on miR-338-3p’s affinity for AGO proteins, but overexpression experiments in Neuro-2a cells found no difference in the isomiRs’ abilities to inhibit targets. Overexpression of miRNAs using artificial mimics increased isomiR levels over 1,000-fold. We might observe differences in isomiR activity with more subtle manipulations. Ideally, we might also inhibit individual isomiRs. However, miRNA inhibitors do not depend on 3′ binding. Thus, without identifying the mechanism underlying miR-338-3p trimming, we do not have a method for conducting loss of function experiments for specific miR-338-3p isomiRs.

Another approach for addressing differences in isomiR function would be to use CLEAR-CLIP to simultaneously sequence miRNA binding sites with the miRNA bound to the target site (Moore et al., 2015). This method has previously been used to examine the effects of 5′ isomiR variants on miRNA targeting (Bjerke and Yi, 2020). However, shifts in the 5′ end of the miRNA often affect the seed sequence (nucleotides 2–7) of the miRNA, which mediate the majority of target interactions. Shifts in the miRNA 3′ end have more subtle effects on mRNA targeting; though CLEAR-CLIP has previously demonstrated that base pairing between an mRNA target and the miRNA 3′ supplemental region, which lies 3′ from the miRNA seed sequence, is extensive *in vivo* and may be critical for fine tuning target specificity (Moore et al., 2015). *In vitro* studies have also demonstrated that miRNA 3′ end length influences interactions between the 3′ supplemental region and mRNA targets, which in turn influences the strength of target inhibition (Sheu-Gruttadauria et al., 2019). Therefore, miRNA 3′ shortening with age might reflect a subtle shift in miRNA targeting efficiency or target identity with age.

Finally, we found that miRNAs that underwent 3′ shortening with age exhibited stronger negative correlations with mRNA and protein targets that decreased with age and stronger positive correlations with targets that increased with age. An important limitation of this analysis is that it was based on predicted targets or targeting data from databases, which might not apply across all biological contexts. However, these patterns in correlation strength were consistent across our studies in the cortex and hippocampus, as well as two independent data sets from mouse and human subjects of different ages. We can only speculate on the significance of these findings though.

### 3.1. Ideas and speculation

As previously stated, miRNAs that underwent 3′ shortening with age exhibited stronger negative correlations with targets that decreased with age and stronger positive correlations with targets that increased with age than miRNAs that did not undergo shortening. GO enrichment analysis of targets within these interactions were associated with neurodevelopment and/or synaptic function across multiple data sets. This suggests miRNA 3′ shortening may be localized within neurons. Notably, when we examined tissues in mice of different ages, we only observed an increase in miR-338-3p 3′ trimming in brain tissues, which is consistent with trimming occurring in neurons. However, this does not rule out miR-338-3p trimming in other cells in the central nervous system. Single cell sequencing of miRNAs in brain would allow us to identify the cells that contribute to 3′ shortening with age, but this technique is not widely available and has not been used to examine isomiR variations in brain to date.

Negative correlations between miRNAs that increase with age and mRNA targets that decrease with age are consistent with our understanding that miRNAs primarily inhibit their mRNA targets. Our observation that miRNAs that undergo shortening exhibit stronger negative correlations in this context suggests either (1) that miRNAs that undergo shortening exhibit more potent inhibition of their targets, (2) that inhibiting mRNA targets promotes miRNA 3′ shortening, or (3) an unidentified outside variable promotes both miRNA shortening and target downregulation.

Our observation that miRNAs that undergo 3′ shortening also exhibit more positive correlations with targets that increase with age is more puzzling. However, a miRNA can only interact with a target that is co-expressed in space and time. Frequently, transcription factors regulate the expression of both miRNAs and their mRNA targets, creating feedforward or feedback loops that finetune mRNA target levels (Tsang et al., 2007). If the transcription of miRNA and target genes are both upregulated during neurodevelopment, the miRNA and its target might exhibit a positive correlation, even while the miRNA is inhibiting that target. A previous publication reported significant miRNA and target co-upregulation during cerebellar, but not lung, development in postnatal mice (Liu and Kohane, 2009). This co-expression was particularly apparent among miRNAs implicated in synaptic transmission. This bears a remarkable resemblance to our finding that upregulated, positively correlated targets were associated with synaptic terms in our cortical data sets, as well as our data mining results from V1 mouse cortex from P10 to P28 (Figure 5) and human prefrontal cortex across the lifespan (Figure 8).

An intriguing possibility is that 3′ shortening affects miRNAs that undergo an increase in mRNA target interactions with age. MiRNAs that undergo 3′ shortening with age have significantly more co-expressed targets in our Combo-Seq data sets, as well as in data mining results from the mouse V1 cortex (Figure 5) and mouse S1 cortex when adult time points were included (Figure 6). MiRNAs that undergo 3′ shortening were more likely to increase with age in most of our analyses, which suggests an increase in their functional importance. Shortening was associated with stronger negative correlations with downregulated targets, which is also consistent with increased targeting. Conversely, transcriptional co-upregulation of mRNA targets would increase the likelihood of an miRNA encountering an mRNA target. Therefore, increased positive correlations with upregulated mRNA targets may also reflect increased target interactions among miRNAs that undergo 3′ shortening with age.

Together, these observations suggest that increased mRNA target interactions may contribute to miRNA 3′ shortening with age. mRNA targets are known to influence miRNA 3′ ends through target directed miRNA degradation (TDMD), which begins with modest 3′ trimming and tailing of miRNAs bound to highly complementary targets (Han and Mendell, 2022). However, miRNAs that underwent 3′ shortening increased with age, suggesting that shortening did not promote degradation. It is also unclear why the 3′ modifications that we observed favored 3′ shortening over 3′ tailing. Further studies are needed to uncover the molecular mechanisms contributing to 3′ end dynamics during neurodevelopment and aging in the mammalian brain.

Notably, our studies focused on wild type mice and neurotypical human subjects. We do not know how or if 3′ end dynamics may be altered in mouse models or human subjects with psychiatric disease. Our data suggest that future studies of miRNA dysregulation in disease should consider changes in miRNA length and sequence, and not just miRNA levels.

## 4. Methods

### 4.1. Animals and tissue collection

For RNA-seq and quantitative proteomics experiments, wild type C57Bl/6J mice of both sexes were used at the following ages in postnatal days (P) ± 2 days: P22, P40, P60, and P120. Tissue was collected from 8 to 9 animals per age. Animals were euthanized by rapid decapitation and the following tissues were collected: cortex (left or right hemisphere, caudal half only), hippocampus, olfactory bulb, dorsal striatum, thalamus (whole), cerebellum, kidney, liver, whole blood (trunk), and testis (male animals only) or ovaries (female animals only). Blood samples were directly collected in tubes provided in the Mouse RiboPure Blood RNA Isolation Kit (Thermo Fisher, AM1951). Brain regions of interest were dissected in cold dissecting ACSF solution containing 125 mM choline Cl, 2.5 mM KCl, 0.4 mM CaCl_2_, 6 mM MgCl_2_, 1.25 mM NaH_2_PO_4_, 26 mM NaHCO_3_, and 20 mM glucose (300–310 mOsm). Tissues were collected in pre-weighed tubes, immediately frozen on dry ice, and stored at −80°C. For experiments with mouse cortex, cortical tissue was further dissected into small pieces, and those pieces were randomly assigned to RNA-seq or quantitative proteomics experiments. For RT-qPCR experiments shown in Supplementary Figure S1B, cortex, hippocampus, and medial geniculate nucleus containing thalamus (MGv) were collected from wild type C57Bl/6J mice of both sexes at the following ages in postnatal days (P) ± 2 days: P22, P40, P60, P90, P120 and P150. No animals from this cohort were used in RNA-seq or quantitative proteomics experiments. The care and use of animals were reviewed and approved by the St. Jude Institutional Animal Care and Use Committee.

### 4.2. RNA isolation and quality control

Total RNA was isolated using the following kits per manufacturers’ instructions: mirVana miRNA Isolation Kit (Thermo Fisher, AM1560; hippocampus, cerebellum, olfactory bulb, thalamus, kidney, kidney, testis, and ovaries), Direct-zol RNA Miniprep Kits (Zymo Research, R2051; cortex), Direct-zol RNA Microprep Kits (Zymo Research, R2062; liver, striatum, and immunoprecipitation experiments), or Mouse RiboPure Blood RNA Isolation Kit (Thermo Fisher, AM1951; whole blood). For experiments in Supplementary Figure S1C, total RNA was isolated using the mirVana miRNA Isolation Kit. With Zymo kits, on-column DNase treatment was performed on all samples. All RNA samples (including samples isolated by Zymo kits) underwent DNase treatment using the DNA-free DNA Removal Kit (Thermo Fisher, AM1906). RNA concentrations and 260/230 and 260/280 ratios were measured by Nanodrop. RNA integrity was assessed in all samples used for RNA-seq by 2100 Bioanalyzer RNA 6000 Nano assay (Agilent) or 4200 TapeStation High Sensitivity RNA ScreenTape assay (Agilent) prior to library generation. For hippocampus RNA samples, RNA concentrations were also assessed using Quant-it RiboGreen RNA Assay Kit (Thermo Fisher, R11490).

### 4.3. Combined mRNA/miRNA sequencing (Combo-Seq)

For RNA-seq experiments, ERCC RNA Spike-in Mix (Thermo Fisher, 4456740, 1 μL of 1:1,000 diluted spike-in mix per sample) and QIAseq miRNA Library QC Spike-in (Qiagen, 331535, 1 μL per sample) were added to 50 ng of total RNA per sample. Libraries were then prepared using the NEXTFLEX Combo-Seq mRNA/miRNA Kit (Perkin Elmer, NOVA-5139-02) per manufacturer’s instructions. Libraries were analyzed for insert size distribution using the 4200 TapeStation D1000 ScreenTape assay (Agilent). Libraries were quantified using the Quant-iT PicoGreen ds DNA assay (ThermoFisher). Paired end 100 cycle sequencing (cortex) or single end 50 cycle sequencing (hippocampus) was performed on a NovaSeq 6000 (Illumina). For paired end sequencing results (cortex), only read 1 data were used in downstream analyses, as the poly(A)-tail added during library preparation creates a poly(T)-stretch at the beginning of read 2 that reduces sequencing accuracy.

### 4.4. Plasmids, miRNA mimics, and oligomers

To generate the psiCHECK-2-*Drd2*-3′UTR and psiCHECK-2-*Aatk*-3′UTR plasmids for luciferase assays, mouse 3′ UTR sequences were amplified from genomic DNA and inserted into the psi-CHECK-2 (Promega, C8021) backbone downstream of the *Renilla* luciferase coding sequence using the NotI and XhoI restriction sites. The mouse *Drd2*-3′UTR sequence was PCR amplified and restriction sites were added using the following primers: 5′-TCGACTCGAGGTCTGCCCCTTGCCTGCACAG-3′ and 5′-TCGAGCGGCC GCCCGTGGGAATGACTCTTGTCAAG-3′. The mouse *Aatk-*3′UTR sequence was similarly amplified using the following primers: 5′-TCGACTCGAGGACCCAGGTTATCCCACCCTTC-3′ and 5′-TCGAGCGGCCGCCTTAGCTTGGTTTCTCTTAAAAAACAAGG-3′.

For experiments in Neuro-2a cells, the following mirVana miRNA mimics (Catalog #4464058) were purchased from Thermo Fisher Scientific: negative control (Negative Control #1, 4464058, no Assay ID), miR-338-3p FL (hsa-miR-338-3p, Assay ID MC10716), miR-338-3p -G (aca-miR-338-3p, Assay ID MC23015), and miR-338-3p -UG (Custom Assay, Assay ID AK20SGT). Except for luciferase assay experiments, mimics were transfected into Neuro-2a cells using Lipofectamine RNAiMAX (Thermo Fisher, 13778150) at a final concentration of 30 nM per well.

To validate miR-338-3p isomiR-specific RT-qPCR primers, the following 5′-phosphorylated RNA oligomers were purchased from IDT: 5′-UCCAGCAUCAGUGAUUUUGUUGA-3′ (miR-338-3p + A), 5′-UCCAGCAUCAGUGAUUUUGUUG-3′ (miR-338-3p FL), UCCAGCAUCAGUGAUUUUGUU-3′ (miR-338-3p -G), and 5′-UCCAGCAUCAGUGAUUUUGU-3′ (miR-338-3p -UG).

### 4.5. Luciferase assays

Neuro-2a cells were transfected at ~50–70% confluence with the following constructs: (1) mirVana miRNA mimic (30 nM, final concentration) and (2) psiCHECK-2 plasmid (0.25 μg/well) encoding *Renilla* and firefly luciferase and containing the mouse *Aatk-*3′UTR, *Drd2*-3′UTR, or no 3′UTR downstream of the *Renilla* coding sequence. Transfections were conducted using Lipofectamine 2000 (Thermo Fisher, 11668030) per manufacturer’s instructions. Approximately 24 h after transfection, luciferase activities were measured using the Dual-Glo Luciferase Assay System (Promega, E2920) and a PHERAstar FSX Microplate Reader (BMG Labtech). Within each well, *Renilla* luciferase activity was normalized to firefly luciferase activity to control for differences in transfection efficiency between wells.

### 4.6. Neuro-2a cell cultures

Neuro-2a cells (ATCC, CCL-131), a mouse neuroblastoma-derived cell line, were cultured in DMEM (Dulbecco’s Modified Eagle Medium, Thermo Fisher Scientific, 11995065) supplemented with 10% FBS (Thermo Fisher Scientific, 16000044) and Penicillin–Streptomycin (Thermo Fisher Scientific, 10378016) at 37°C in a humidified atmosphere of 5% C0_2_ and ambient oxygen. Cultures were verified to be free of mycoplasma contamination using Universal Mycoplasma Detection Kit (ATCC, 30–1,012 K). For all experiments using Neuro-2a cells, the “n” in the figure legend represents the number of biological replicates for each experiment. Within biological replicates, all conditions were represented in triplicate, and the mean measurement was used for statistical analysis.

### 4.7. Microarray data analysis (mRNA)

Log_2_-transformed microarray data for GSE17757 were retrieved using GEOquery (v2.62.2) (Sean and Meltzer, 2007) and analyzed in R. Data were subset to only include human subjects and averaged across technical replicates. Data were normalized between subjects using limma (v3.50.3) (Ritchie et al., 2015). DE analysis and gene clustering were performed using maSigPro (v1.66.0) (Nueda et al., 2014).

### 4.8. Microarray data analysis (miRNA)

MiRNA microarrays were prepared and data were extracted as previously described (Chun et al., 2017). After removing probes with low signal intensity (less than one for all samples among comparison groups), quantile normalization scaling against nine positive control probes included on the microarray was applied among all samples. Each probe was then summarized by averaging all duplicate spots for a single normalized intensity value. The Student’s *t*-test was used to determine the statistically significant difference between sets of replicates from different experimental groups (4-mo vs. 2-mo samples), and *p*-values were corrected for multiple comparisons using the false discovery rate (FDR) method. The miRNA was considered significantly differentially expressed with FDR < 0.05.

### 4.9. AGO-RNA co-immunoprecipitation (AGO-RIP)

Whole hippocampus from 4 male WT type C57Bl/6J was collected at P119 or P120, frozen on dry ice, and stored at −80°C until ready for use. Tissue was then mechanically lysed in 800 μL of NP-40 lysis buffer per animal, containing phosphatase, protease, and RNase inhibitors: 50 mM Tris–HCl (Sigma, T3038), 1% v/v IGEPAL CA-630 (Sigma, I3021), 150 mM NaCl, 1X cOmplete mini (Roche, 11836153001), 1X phosSTOP, (Sigma, 4906845001), and 0.2 U/μL SUPERase In RNase Inhibitor (Thermo Fisher, AM2696). Protein concentrations were measured by Pierce BCA Protein Assay Kit (Thermo Fisher, 23225). Samples were then prepped for 2 IPs per subject: one with unconjugated beads and one with AGO-antibody conjugated beads, as follows. Lysate containing 1,500 μg of protein per animal per IP with volumes adjusted to 400 μL with NP-40 lysis buffer was pre-cleared by incubating with 50 μL of Pierce protein A/G magnetic beads (Thermo Fisher, 88836), rotating for 30 min at 4°C. Magnetic beads were then pelleted by a magnetic stand, and pre-cleared lysate was added to unconjugated magnetic beads or magnetic beads that had been pre-conjugated with 6 μg of anti-AGO antibody (mouse anti-Ago2, Abcam, clone 2E12-1C9) per sample. Samples were incubated by rotating for 5 h at 4°C. Beads were then pelleted by a magnetic stand, and flow through samples were collected from the supernatant for RNA and western blot experiments. Beads were washed 4 times with NP-40 buffer to remove unbound protein and miRNAs. Beads were then split for downstream RNA (90% of sample) and western blot assays (10% of sample). RNA was eluted from beads by mixing in Trizol. Protein was eluted in 1X NuPAGE LDS Sample Buffer (Thermo Fisher, NP0007) by heating for 10 min at 70°C. β-mercaptoethanol (2.5%) was added, and samples were heated for 10 min at 70°C before loading for SDS-PAGE.

### 4.10. Mass spectrometry validation of AGO-IP

For mass spectrometry validation experiments, IP was performed as described above but with the following modifications. The IP was performed with 2 mg of protein derived from the frontal cortex of a male P60 mouse, 40 μL of magnetic beads, and 10 μg of antibody. Also, 100% of the sample was collected for protein experiments. 2-mercaptoethanol was added to a final concentration of 2.5%, then proteins were resolved by SDS-PAGE. Silver staining was performed using the Pierce Silver Stain kit (Thermo Fisher, 24612). Bands at 97 kDa were extracted from the pulldown fractions of the AGO-IP and no antibody control IP, destained using the SilverQuest Staining Kit (Thermo Fisher, LC6070), and analyzed according to a previously optimized In-gel trypsin digestion followed by the nanoscale LC–MS/MS protocol (Xu et al., 2009). The MS/MS data were searched against a Mouse FASTA target-decoy database and filtered to reduce protein false discovery rate to below 1% (as described below, see quantitative proteomics methods).

### 4.11. Reverse transcription-quantitative PCR (RT-qPCR)

Reverse transcription for mRNA detection was performed using iScript Reverse Transcription Supermix (Bio-Rad, 1708841) per manufacturer’s instructions. Reverse transcription for miRNA (except for LNA probes, see below), U6 snRNA, snoRNA234, or 5.8S rRNA detection was performed using the miRNA 1st-Strand cDNA Synthesis Kit (Agilent, 600036). qPCR was performed using the SYBR Green PCR Master Mix (Thermo Fisher, 4309155) and a C1000 Touch Thermal Cycler (Bio-Rad). Primer sequences are provided in Table 2.

**Table 2:** RT-qPCR primer sequences shown 5′–3′.

Target	Forward primer	Reverse primer
5.8S rRNA	GGTGGATCACTCGGCTCGT	GCAAGTGCGTTCGAAGTGTC
*Aatk* mRNA	ATGCTGGCCTGCCTGTGTTGT	AGGGGCAGGACATACACATCGG
*Gapdh* mRNA	CATCACTGCCACCCAGAAGACTG	ATGCCAGTGAGCTTCCCGTTCAG
miR-338-3p FL	TCCAGCATCAGTGATTTTGTTG	Universal
miR-338-3p −1spec	TCCAGCATCAGTGATTTTGTTA	Universal
miR-338-3p −2spec	TCCAGCATCAGTGATTTTGTAA	Universal
miR-338-3p -1 nt	TCCAGCATCAGTGATTTTGTT	Universal
miR-338-3p -2 nt	TCCAGCATCAGTGATTTTGT	Universal
*Nova1* mRNA	GCCAGTACTTTCTAAAGGTTCTCA	AGTGGCTCCAGTTTCTTTTTG
snoRNA234	TTAACAAAAATTCGTCACTACCA	Universal
U6 snRNA	CGCTTCGGCAGCACATATAC	TTCACGAATTTGCGTGTCAT

For locked nucleic acid (LNA) RT-qPCR, reverse transcription was performed using miRCURY LNA RT Kit (Qiagen, 339340). qPCR was performed using miRCURY LNA SYBR Green PCR Kit (Qiagen, 339346) and a C1000 Touch Thermal Cycler. MiR-338-3p was detected using the hsa-miR-338-3p miRCURY LNA miRNA PCR Assay (GeneGlobe ID: YP00204719).

For RT-qPCR experiments, data were analyzed using the 2^−ΔΔCq^ method [previously known as the 2^−ΔΔCt^ method, first described in the Applied Biosystems User Bulletin 2 (P/N 4303859)] (Livak and Schmittgen, 2001).

### 4.12. SDS-PAGE and Western blots

Cells or tissues were lysed in NP-40 lysis buffer, containing phosphatase and protease inhibitors: 50 mM Tris–HCl (Sigma, T3038), 1% v/v IGEPAL CA-630 (Sigma, I3021), 150 mM NaCl, 1X cOmplete mini (Roche, 11836153001), and 1X phosSTOP, (Sigma, 4906845001). Protein concentrations were measured by the Pierce BCA Protein Assay Kit (Thermo Fisher, 23225). 8 μg of protein per sample was diluted in the NuPAGE LDS Sample Buffer (Thermo Fisher, NP0007) (1X final concentration) and β-mercaptoethanol (2.5% final concentration) and heated 10 min at 70°C before loading for SDS-PAGE.

Proteins were resolved by SDS-PAGE using NuPAGE Bis-Tris gels (Thermo Fisher) and transferred to PVDF (0.2 μm, NOVA1 western blots) or nitrocellulose membrane (0.45 μm, AGO2 western blots). Transferred proteins were stained using Revert 700 Total Protein Stain (Li-Cor) and imaged on an Odyssey CLX (LI-COR). Staining was then reversed, and membranes were blocked using Intercept Blocking Buffer (LI-COR, 927–70001) for 15 min at room temperature. Blots were incubated overnight in primary antibody diluted in a 1:1 mixture of block and PBS-Tween (0.1%). Washes were performed in PBS-Tween (0.1%). Secondary antibodies were diluted in a 1:1 mixture of block and PBS-Tween (0.1%) and incubated with blots for 1 h at room temperature (or 2 h for NOVA1 western blot). Blots were imaged on an Odyssey CLX. In some cases, western blot was then performed for ACTB (see figure legends). Band densitometry was performed in Fiji (ImageJ) (Schindelin et al., 2012). Data were normalized as described in the figure legend.

The following primary antibodies were used: rabbit anti-NOVA1 (1:500; Thermo Fisher, PA5-95571), mouse anti-ACTB/β-actin (1:10,000; clone AC-15; Santa Cruz, sc-69,879), rabbit anti-AGO2 (1:500; Abcam, ab32381), rabbit anti-DGCR8 (1:1000; Abcam, ab191875), and rabbit anti-DICER (1:2000; Novus, NBP1-06520). The following secondary antibodies were used: donkey anti-mouse IRDye 800CW (1:2000; LI-COR, 926–32,212) and donkey anti-rabbit IRDye 800CW (1:20,000 for NOVA1; 1:10,000 for AGO2; LI-COR, 925–32213).

### 4.13. ExceRpt pipeline for RNA-seq analysis

All Combo-Seq and miRNA sequencing files were processed using the exceRpt Docker image installed on an institutional server (Rozowsky et al., 2019). Mouse and human sequencing files were aligned to exceRpt mm10 and hg38 reference genome files, respectively. For Combo-Seq libraries, ERCC mRNA and Qiagen miRNA spike-in reads were aligned to a custom index generated using bowtie 2 (v2.3.5.1) (Langmead and Salzberg, 2012) from sequences provided by the manufacturers. Minimum read length and STAR alignment minimum match length were set to 15. For Combo-Seq libraries, 4 random nucleotides were trimmed from the 5′ end of each read. The following 3′ adapter sequences were trimmed: AAAAAAAAAA (Hippocampus Combo-Seq), AAAAAAAA (Cortex Combo-Seq), TGGAATTCTCGGGTGCCAAGG (mouse V1 and S1 cortex, human cortex GSE59286), and TCGTATGCCGTCTTCTGCTTGT (human cortex GSE18012). ExceRpt analysis was performed for each sample individually. Then outputs were combined within studies using the mergePipelineRuns.R script.^1^

### 4.14. QuagmiR IsomiR analysis

IsomiR analysis was performed using QuagmiR (Bofill-De Ros et al., 2019). For endogenous miRNA analysis, fastq files (*.fastq.clipped.trimmed.filtered.noRiboRNA.fastq.gz) were pulled from the exceRpt pipeline output. For spike-in analysis, calibrator-mapped reads were extracted from bam files (*.fastq.clipped.trimmed.filtered.calibratormapped.bam) and converted to fastq files using SAMtools (v1.11) (Danecek et al., 2021). Reads were filtered for length (length > =17 and < =26 nucleotides), and the filtered fastq files were uploaded to the Cancer Genomics Cloud. Analysis was performed using the QuagmiR cloud application with the following non-default settings: destructive motif pull = TRUE, 5′ end distance filtering threshold = 3, 3′ end distance filtering threshold = 5, and minimum reads = 10. For endogenous miRNA alignments, custom motif files were generated as described at https://github.com/Gu-Lab-RBL-NCI/QuagmiR (Supplementary Data Sheets 2, 3). Motif length was set to 15 nucleotides, starting at position 4. Files were manually edited to add some highly abundant miRNAs missing from the original files. For Qiagen miRNA spike-in alignment, motifs were generated from nucleotides 4 through 15 in the spike-in sequence (Supplementary Data Sheet 4).

### 4.15. Principal component analysis (PCA) and outlier identification

PCA was performed using 3′ end scores, normalized counts, or other expression data (see figure legends) using the prcomp() function from stats (v3.6.2) in R. For RNA-seq expression data, variance stabilizing transform, vst(), normalized data from DESeq2 were used for PCA. Graphs were generated using the fviz_pca_ind() function from factoextra (v1.0.7) and customized using ggplot2 (v3.3.6). Outliers were identified visually and removed prior to generating the graphs shown in figures. These outliers are identified in figure legends for PCA plots.

### 4.16. 3′ end regression analysis

3′ ERA was performed in R using files generated by QuagmiR (*.group_output.isomir.sequence_info.tsv). Analysis was restricted to miRNAs detected at a mean of 10 reads per million miRNA reads. First, the end position for each isomiR within each sample was calculated by subtracting the LEN_TRIM column from the LEN_TAIL column. The isomiR end position was then multiplied by the frequency of the isomiR within that sample, with the frequency corresponding to the numerical equivalent of the RATIO in the QuagmiR output file. These weighted end positions were then summed over all isomiRs for each miRNA to generate a 3′ end score for each miRNA within each sample. PCA was performed using these 3′ end scores and outliers (if any) were removed. Following outlier removal, two technical replicates remained for the D34 subject in GSE18012. A mean 3′ end score was calculated for each miRNA and used for downstream analyses, rather than individual values from technical replicates. Linear regression analysis was then performed for each miRNA using the lm() function in R using 3′ end scores and the following models: ~ Age + Batch + Sex (Combo-Seq studies), ~ PND (mouse V1 cortex), ~ log(PND) + Sex + Layer (mouse S1 cortex, including P180 samples), ~ PND + Sex + Layer (mouse S1 cortex, excluding P180 samples), ~ log(Days) + Sex + Diagnosis (human cortex GSE59286), and ~ log(Days) (human cortex GSE18012). Here, Age, Days, and PND correspond to the subject age in days (human) or postnatal days (PND) (mouse). Estimates and *p*-values corresponding to the Age/Days/PND coefficient were compiled, and *p*-values were corrected for multiple comparisons using the FDR method. MiRNA 3′ ends were considered “extended” with age if Estimate >0 and FDR < 0.05. MiRNA 3′ ends were considered “shortened” with age if Estimate <0 and FDR < 0.05.

To determine the source of age-associated changes in 3′ end length, 3′ ERA was repeated for each type of modification that miRNA 3′ end can undergo, i.e., 3′ trimming or 3′ tailing with each RNA nucleotide. For 3′ trimming, only LEN_TRIM was used. For 3′ tailing, stringr (v1.4.1) was used to calculate the number of occurrences of each nucleotide in LEN_TAIL. Downstream analyses were then performed as described above.

### 4.17. MiRNA read position coverage

MiRNA read position coverage was calculated using files generated by QuagmiR (*.group_output.isomir.nucleotide_dist.tsv). Reads were summed by nucleotide position (NT_POSITION) within each sample, and then normalized to the total number of reads for each sample. Notably, NT_POSITION represents the position of each nucleotide relative to the canonical 5′ end nucleotide for each miRNA, and not the position of the nucleotide within a sequencing read. Graphs show the mean coverage at each nucleotide position by PND.

### 4.18. Differential expression analysis (RNA-seq)

For analyses in Figure 1, exceRpt-derived read counts were used for all RNAs. For all other analyses, QuagmiR-derived read counts were used for miRNAs (endogenous and spike-in), and exceRpt-derived counts were used for all other RNAs, including mRNA-like spike-in RNAs. Spike-in and endogenous read counts were combined in a single matrix. For experiments in which mRNAs and miRNAs were sequenced separately (i.e., non-Combo-Seq experiments), differential expression analyses were also conducted separately. All genes containing fewer than 10 total counts were removed before data normalization. Differential expression analyses were performed in DESeq2 (v1.34.0) (Love et al., 2014) using the following designs: ~ Batch + Sex + Age (Combo-Seq), ~ PND (mouse V1 cortex), ~ Layer + Sex + log(PND) (mouse S1 cortex, including P180 samples), ~ Layer + Sex + PND (mouse S1 cortex, excluding P180 samples), and ~ Batch + log(Days) (human cortex GSE18012). For V1 cortex, the default Wald test was used for differential expression analysis. For time series experiments containing more than 2 time points, differential expression analysis was performed using the likelihood ratio test (LRT), with a reduced model containing any non-age variables. Rlog() transformed data for differentially expressed genes (defined as adjusted value of *p* < 0.05) were extracted and used for clustering analysis. Clustering analysis was performed using the degPatterns() function from DEGreport (v1.30.3) (Pantano, 2022). For clustering analysis, age was treated as a categorical variable, e.g., the PND variable in Combo-Seq experiments. For human cortex GSE18012, subjects were divided into age groups as follows: <1 year (infant), 1–12 years (child), 13–18 years (adolescent), 19–29 years (young adult), 30–64 years (adult), and 65+ years (elderly).

### 4.19. Quantitative proteomics by tandem mass tag mass spectrometry (TMT-MS)

Quantitative proteome profiling was based on a previously optimized protocol (Yu et al., 2021). Briefly, mouse cortex tissue samples in 8 M urea lysis buffer were lysed by pulse sonication, and protein amounts were quantified. Approximately, 100 μg proteins for each TMT channel were digested with Lys-C (Wako, 1:100 w/w) at 21°C for 2 h, diluted to reduce urea to 2 M, and further digested with trypsin (Promega, 1:50 w/w) at 21°C overnight. The protein digests were acidified (trifluoroacetic acid to 1%), desalted with Sep-Pak C18 cartridge (Waters), and dried by Speedvac. Each sample was resuspended in 50 mM HEPES (pH 8.5) for TMT labeling, and equally pooled, and desalted for the subsequent fractionation by offline basic pH reverse phase LC (a Waters XBridge C18 column, 3.5 μm particle size, 4.6 mm × 25 cm, 180 min gradient). Each of these fractions (~80 concatenated fractions) was analyzed by the acidic pH reverse phase LC–MS/MS (a CoAnn 75 μm × 30 cm column, packed with 1.9 μm C18 resin from Dr. Maisch GmbH, 80 min gradient), interfaced with Orbitrap Fusion MS (Thermo Fisher). MS settings included MS1 scans (410–1,600 m/z, ~60,000 resolution, 1 × 106 AGC, and 50 ms maximal ion time) and 20 data-dependent MS2 scans (fixed first mass of 120 m/z, 60,000 resolution, 1 × 105 AGC, ~100 ms maximal ion time, HCD, 36% normalized collision energy, ~1.0 m/z isolation window with 0.2 m/z offset, and ~ 15 s dynamic exclusion).

The MS/MS data were searched against a Mouse FASTA database (concatenated target-decoy, version 2022.04.22) using the tag-based hybrid JUMP search engine (Wang et al., 2014). Search parameters included mass tolerance of 20 ppm for precursor ions and MS/MS ions, fully tryptic, static mass shift for the TMT tags (+304.2071453) and carbamidomethyl modification of 57.02146 on cysteine, dynamic mass shift for Met oxidation (+15.99491), maximal missed cleavage (*n* = 2). All matched MS/MS spectra were filtered by mass accuracy and matching scores to reduce protein FDR to about 1%, based on the target-decoy strategy (Peng et al., 2003). TMT-based quantification was performed based on our previous method (Niu et al., 2017). Data are provided in Supplementary Table S1.

### 4.20. Differential expression analysis (quantitative proteomics)

Protein intensities obtained from quantitative proteomics were log_2_ transformed and analyzed in limma. The design matrix was built with the formula ~0 + PND + Batch. Contrasts were made between sequential age groups: P40-P22, P60-P40, and P120-P60. Differential expression analysis was performed using the limma-trend approach. Adjustment for multiple hypothesis testing was performed using decideTests() with method = “global.” Clustering analysis was then performed with proteins differentially expressed (adjusted value of *p* < 0.05) in any of the age group comparisons using the degPatterns() function from DEGreport.

### 4.21. MiRNA:target correlation analysis

For miRNA targeting analysis, we limited our analysis to miRNAs detected at >10 reads per million and targets that met inclusion criteria for our differential expression analyses in each data set. Targeting predictions and validated target interactions were acquired from TargetScan (v8.0) (McGeary et al., 2019), miRNet (v2.0) (Chang et al., 2020), and miRDB (v6.0) (Liu and Wang, 2019; Chen and Wang, 2020). For miRNet, we limited the interactions to those identified *via* HITS-CLIP. Interactions were then filtered to only include those identified by 2 or more tools. We then calculated Pearson correlations across all samples for each miRNA and target using normalized expression data (see differential expression analysis details) and the cor.test() function from stats (v3.6.2) in R.

### 4.22. Gene ontology term enrichment analysis

GO term enrichment analysis was performed using g:Profiler (v.e106_eg53_p16_65fcd97) (Raudvere et al., 2019). Gene selection criteria for each analysis are described in figure legends. For all analyses, a custom background was uploaded containing genes detected in the data set of interest. When *p*-values were available for a comparison, genes were ordered by *p*-value and the “Ordered query” option was selected. Results were filtered by term size, and redundant terms were removed to simplify graphing.

### 4.23. Graphing and statistics

Except for heatmaps and PCA plots, all graphs were generated using ggplot2 (v3.3.6) (Wickham, 2016) in R (v4.1.2). All statistics were calculated in R as described in figure legends and/or methods. For hypothesis testing, parametric tests (e.g., ANOVA, t-test) were used when data were normally distributed and did not contain extreme outliers, while non-parametric tests (e.g., Kruskal-Wallis, Wilcox Test) were used for data sets that did not meet these requirements. Normality was tested using Shapiro’s test, while extreme outliers were identified using the identify_outliers() function in R, which uses the boxplot method for outlier identification.

## Data availability statement

The datasets presented in this study can be found in online repositories. The names of the repository/repositories and accession number(s) can be found at: https://www.ncbi.nlm.nih.gov/, PRJNA935727; http://www.proteomexchange.org/, PXD040229.

## Ethics statement

The animal study was reviewed and approved by the St. Jude Institutional Animal Care and Use Committee.

## Author contributions

KT and SZ: conceptualization. KT, AV, SE, AM, Y-DW, and TL: experiments and data analysis. JP: methodology. SZ: resources and funding. All authors have read and agreed to the published version of the manuscript.

## Funding

This work was supported in part by the National Institutes of Health (NIH) grants R01 DC012833 and R01 MH097742 (SZ) and by the American Lebanese Syrian Associated Charities (ALSAC). The funding sources had no role in study design, data collection, data analysis, decision to publish, or preparation of the manuscript. The content is solely the responsibility of the authors and does not necessarily represent the official views of the NIH or other granting agencies.

## Conflict of interest

The authors declare that the research was conducted in the absence of any commercial or financial relationships that could be construed as a potential conflict of interest.

## Publisher’s note

All claims expressed in this article are solely those of the authors and do not necessarily represent those of their affiliated organizations, or those of the publisher, the editors and the reviewers. Any product that may be evaluated in this article, or claim that may be made by its manufacturer, is not guaranteed or endorsed by the publisher.

## Footnotes

^1^ https://github.com/gersteinlab/exceRpt
