# Supplementary material for: MicroRNA 3′ ends shorten during adolescent brain maturation
Source: Front Mol Neurosci. 2023 Apr 14;16:1168695. doi: 10.3389/fnmol.2023.1168695 (PMC10140418; doi:10.3389/fnmol.2023.1168695)
Supplement: Supplementary file 1 [file Data_Sheet_1.pdf]

# Supplementary Figures

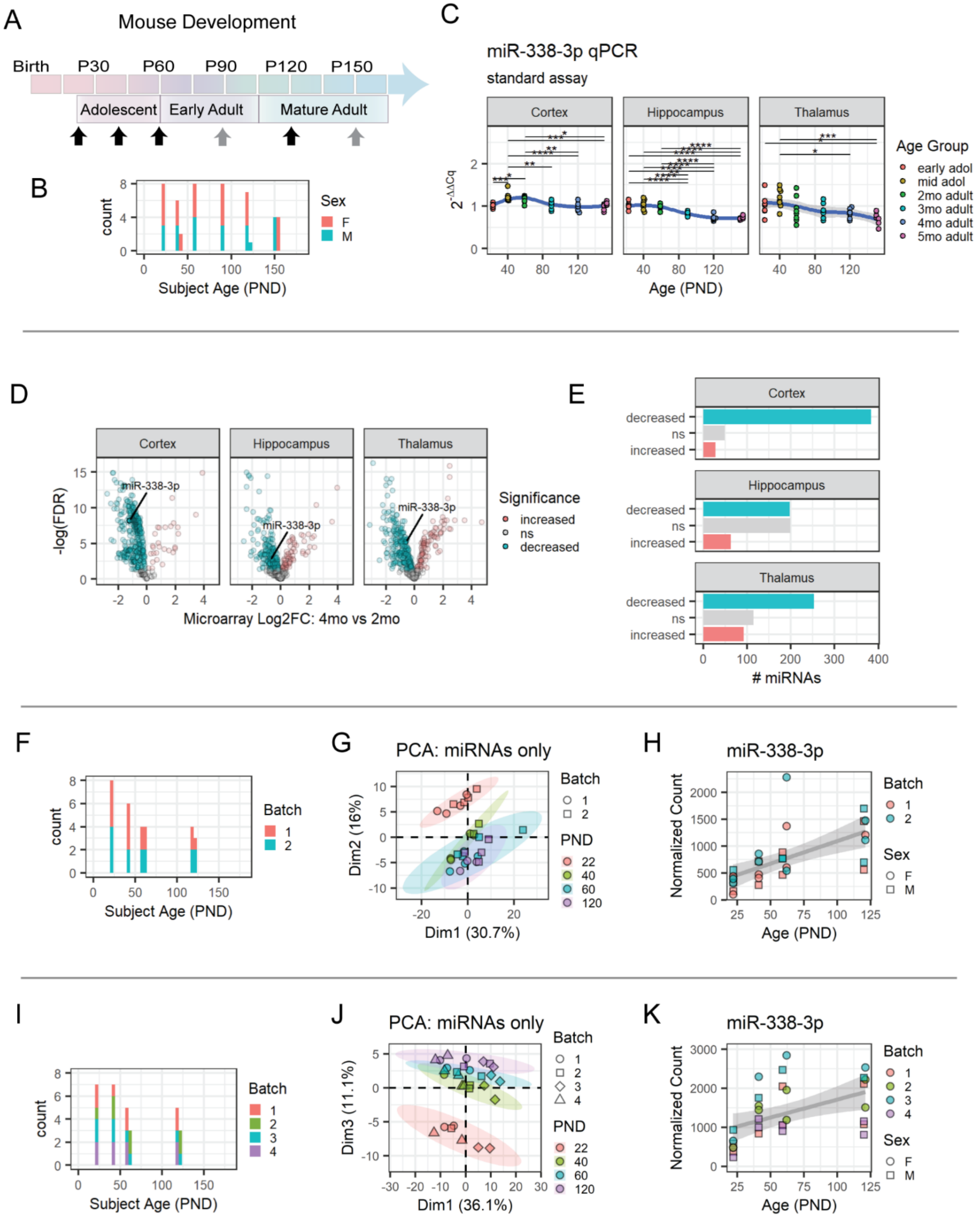


Figure S1

MiR-338-3p appears to decrease with age when detected by standard RT-qPCR assay or miRNA microarray but increases with age when detected by Combo-Seq.

A) Timeline of mouse development with time points for RT-qPCR indicated by arrows. Black arrows indicate time points that were also used for Combo-Seq experiments.
B) Sample age and sex distribution for RT-qPCR data in C.
C) MiR-338-3p levels decrease with age relative to sno234 when detected by standard RT-qPCR assay in mouse cortex, hippocampus, and thalamus (MGv). The forward primer for this assay had the sequence TCCAGCATCAGTGATTTTGTTG. One way ANOVA: p(Cortex) = 1.10e-5, p(Hippocampus) = 1.52e-12, p(Thalamus) = 2.04e-3. Bonferroni adjusted p-values for pairwise comparisons: *p < 0.05, **p < 0.01, ***p < 0.001, ****p < 0.0001. Non-linear trends were fit using the geom_smooth() function in R, with method set to “loess”.
D) Volcano plots summarizing miRNA microarray DE results. MiR-338-3p significantly decreased between 2 and 4 months of age in all tissues.
E) MiRNA microarray DE results summary. The majority of miRNAs decreased with age (relative to spike-in control RNAs) in cortex and thalamus (MGv). In hippocampus, almost 200 miRNAs decreased with age.
F) Sample age and library batch information for mouse cortex samples used in Combo-Seq experiments (not including outliers).
G) PCA was conducted on miRNA expression data from mouse cortex. Samples clustered approximately by age in PND. Two samples (from subjects B-5 and B-6) were identified as outliers and removed from downstream expression analyses based on PCA results (not shown).
H) MiR-338-3p increased with age in mouse cortex when detected by Combo-Seq. Association between age and miRNA levels was assessed by LRT in DESeq2. Estimate (Age) = 0.017068327, p(Age) = 0.000205874. Trend lines were fit using the geom_smooth() function in R, with method set to “lm”.
I) Sample age and library batch information for mouse hippocampus samples used in Combo-Seq experiments.
J) PCA was conducted on miRNA expression data from mouse hippocampus. Samples clustered approximately by age in dimension 3. No samples were identified as outliers based on PCA results.
K) MiR-338-3p increased with age in mouse hippocampus when detected by Combo-Seq. Association between age and miRNA levels was assessed by LRT in DESeq2. Estimate (Age) = 0.011025274, p(Age) = 0.012168761. Trend lines were fit using the geom_smooth() function in R, with method set to “lm”.

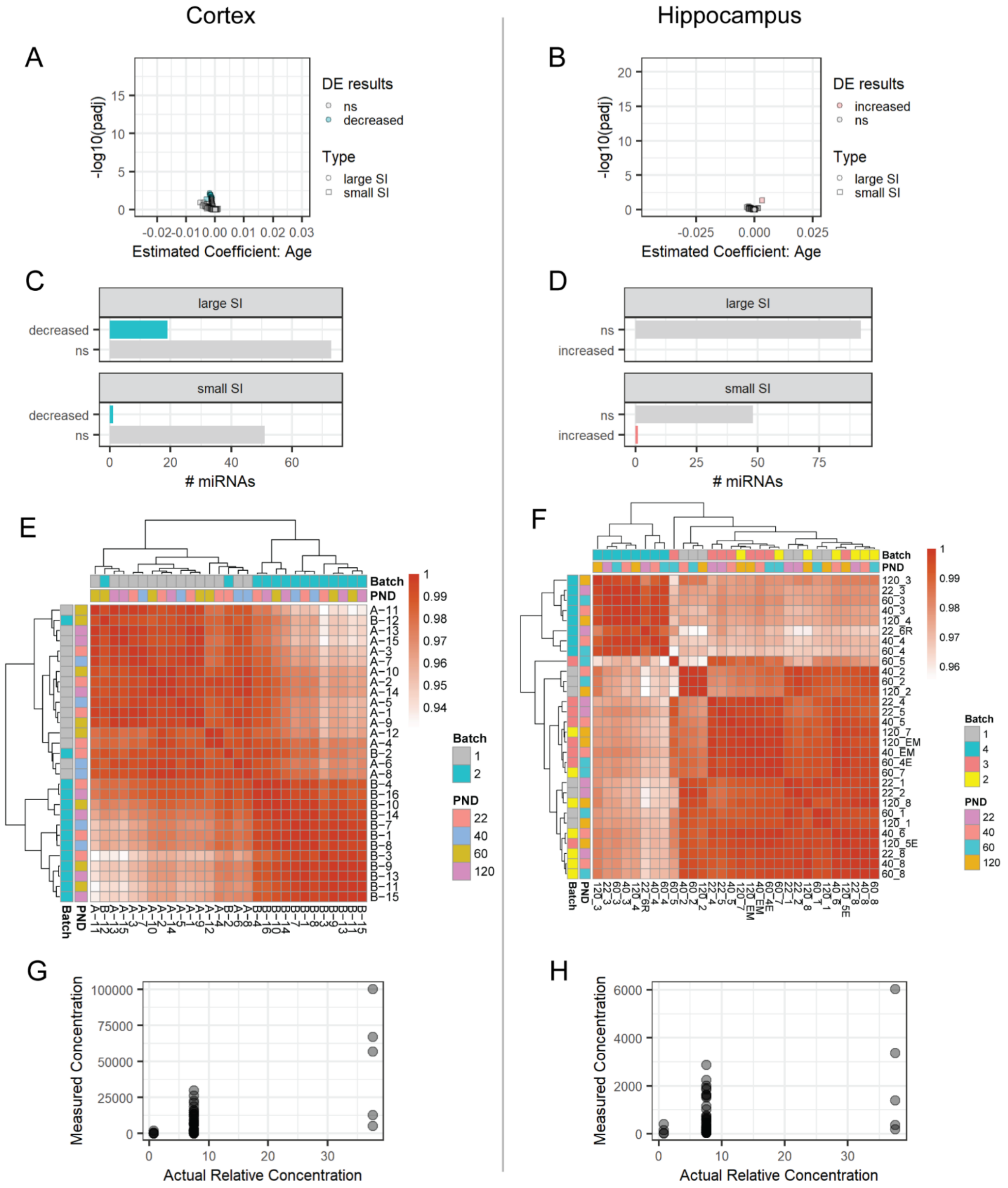


**Figure S2**

Spike-in detection summary for cortex and hippocampus Combo-Seq data sets

A) Volcano plot summarizing DE results for spike-in RNAs in cortex Combo-Seq libraries.

B) Volcano plot summarizing DE results for spike-in RNAs in hippocampus Combo-Seq libraries.
C) DE results summary for spike-in RNAs in cortex libraries. Most spike-in RNAs were correctly detected as not differentially expressed with age. However, some artificially appeared to decrease with age, suggesting our normalization strategy is slightly under-detecting increases in endogenous RNAs with age.
D) DE results summary for spike-in RNAs in hippocampus libraries. Most spike-in RNAs were correctly detected as not differentially expressed with age.
E) Spike-in counts were highly correlated across samples in cortex libraries. Heatmap color scale represents the between-sample Pearson correlation coefficient, r. Samples clustered by batch, not age, suggesting that technical differences between library batches underlie variation in spike-in detection, not biological differences between samples. Overall, results suggest that Combo-Seq is suitable for comparisons of RNA levels between samples.
F) Spike-in counts were highly correlated across samples in hippocampus libraries. Heatmap color scale represents the between-sample Pearson correlation coefficient, r. Samples clustered by batch, not age, suggesting that technical differences between library batches underlie variation in spike-in detection, not biological differences between samples. Overall, results suggest that Combo-Seq is suitable for comparisons of RNA levels between samples.
G) Detected small RNA spike-in concentration in cortex libraries as a function of the actual concentration data provided by the manufacturers. Each point represents one small RNA spike-in. Overall, the detected and actual concentrations were poorly correlated, suggesting Combo-Seq read counts do not accurately reflect relative miRNA concentrations within samples.
H) Detected small RNA spike-in concentration in hippocampus libraries as a function of the actual concentration data provided by the manufacturers. Each point represents one small RNA spike-in. Overall, the detected and actual concentrations were poorly correlated, suggesting Combo-Seq read counts do not accurately reflect relative miRNA concentrations within samples.

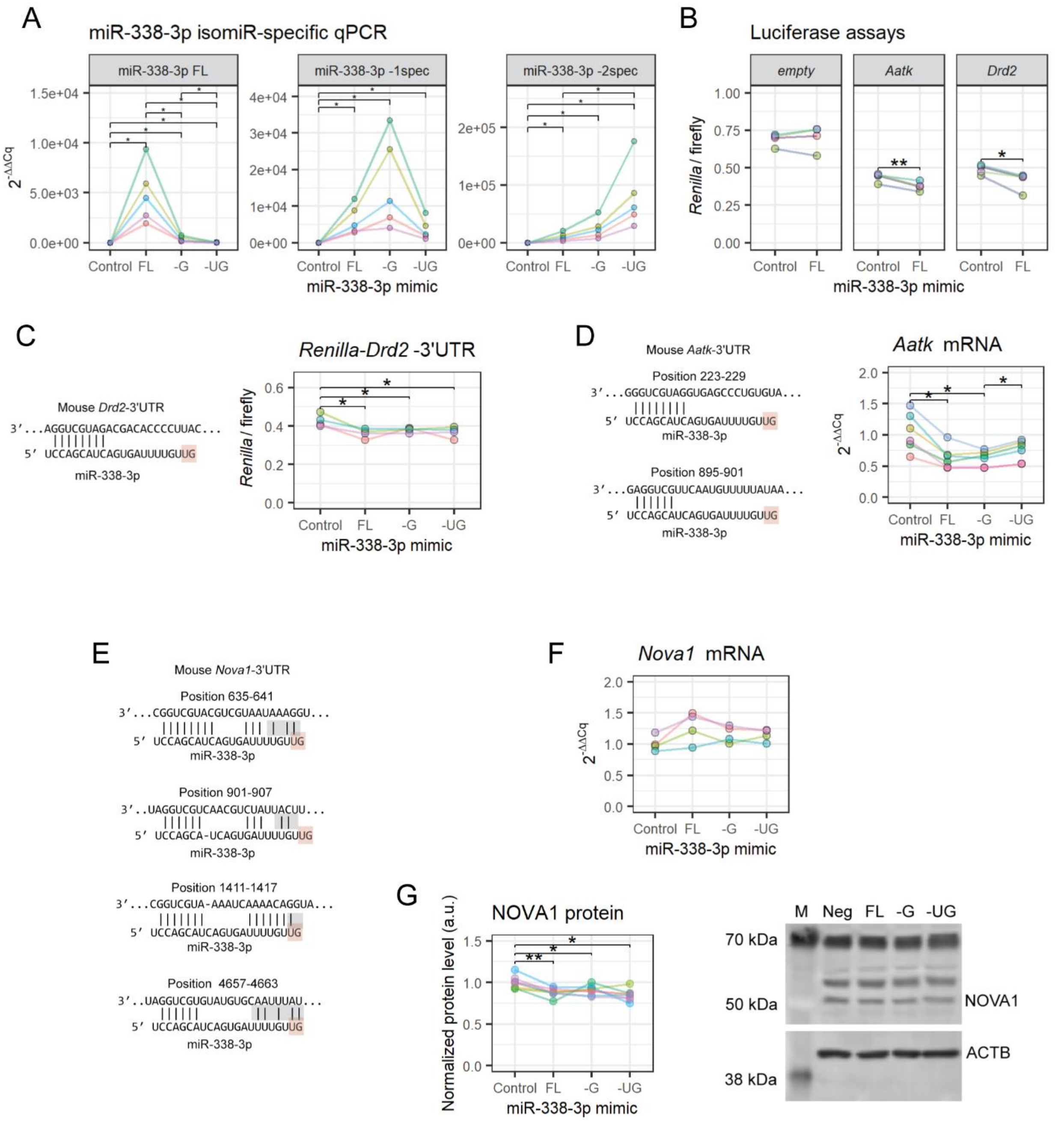


**Figure S3**

MiR-338-3p 3′ trimmed isomiRs inhibit targets when overexpressed in Neuro-2a cells

A) MiR-338-3p isomiR mimics (x-axis) were transfected into Neuro-2a cells and detected by isomiR-specific RT-qPCR assay. Each panel represents a different primer used for isomiR detection. Mimics successfully overexpressed their target isomiRs relative to a negative control mimic. Kruskal Wallis: p(FL) = 0.000536, p(-1spec) = 0.00314, p(-2spec) = 0.000994. Holm adjusted p-values for Wilcox test pairwise comparisons: *p < 0.05. N = 5.

B) Luciferase assays using miR-338-3p FL or negative control mimic in Neuro-2a cells. Each panel represents a 3′UTR sequence downstream of the *Renilla* luciferase coding

sequence. MiR-338-3p FL mimic inhibited the reporters containing the *Aatk*- or *Drd2*-3′UTR, consistent with reports that miR-338-3p targets these mRNAs, but not the reporter construct lacking a target 3′UTR. Two way ANOVA: p(3′UTR) = 1.19e-06, p(mimic) = 4.5e-02, p(interactive) = 1.3e-02. Holm adjusted p-values for t-test pairwise comparisons: *p< 0.05, **p< 0.01. N = 4.

C) MiR-338-3p isomiR mimics inhibit the *Drd2* luciferase reporter to a similar degree in Neuro-2a cells. One way ANOVA: p = 0.004. Holm adjusted p-values for t-test pairwise comparisons: *p< 0.05. N = 4. The predicted target site for miR-338-3p is shown to the left. The 3′ end of miR-338-3p is not predicted to interact with this target site.

D) MiR-338-3p isomiR mimics inhibit endogenous *Aatk* mRNA to a similar degree in Neuro-2a cells. Data was normalized to *Gapdh* mRNA. One way ANOVA: p = 3.69e-05. Holm adjusted p-values for t-test pairwise comparisons: *p< 0.05. N = 6. The predicted target sites for miR-338-3p are shown to the left. The 3′ end of miR-338-3p is not predicted to interact with either target site.

E) MiR-338-3p predicted target sites in the *Nova1*-3′UTR. Base pairing events predicted by TargetScan are shown in white. Additional possible interactions are shown in gray. Notably, at several sites, nucleotides that are trimmed in the -G and/or -UG isomiRs are predicted to interact with the target site.

F) MiR-338-3p isomiR mimics do not affect endogenous *Nova1* mRNA levels. Data were normalized to *Gapdh* mRNA. One way ANOVA: p = 0.024. However, none of the pairwise t-test comparisons were significant. N = 4.

G) MiR-338-3p isomiR mimics decrease endogenous NOVA1 protein levels. Data were normalized to ACTB (i.e., β-actin). Representative blots are shown to the right. One way ANOVA: p = 0.002. Holm adjusted p-values for t-test pairwise comparisons: *p< 0.05, **p< 0.01. N = 8.

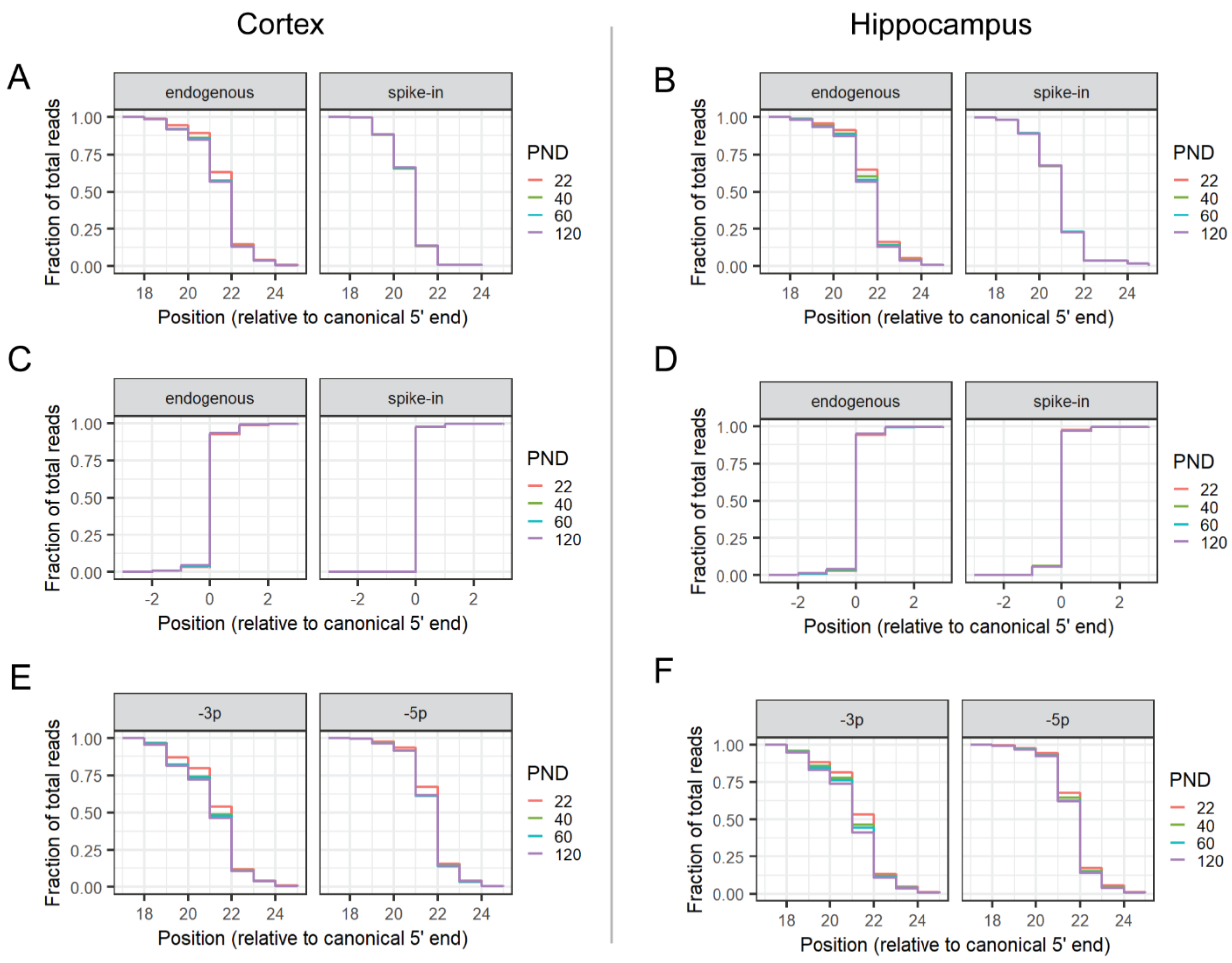


**Figure S4**

Shortening occurs at the 3′ end of endogenous -3p and -5p miRNAs with age.

A) Sequencing coverage at nucleotides 17-25 relative to the canonical 5′ end of endogenous and spike-in RNAs in cortex Combo-Seq libraries. Coverage decreases at positions 20-22 of endogenous miRNAs, but not small spike-in RNAs, between P22 and P120, consistent with 3′ shortening with age.

B) Sequencing coverage at nucleotides 17-25 relative to the canonical 5′ end of endogenous and spike-in RNAs in hippocampus Combo-Seq libraries. Coverage decreases at positions 20-22 of endogenous miRNAs, but not small spike-in RNAs, between P22 and P120, consistent with 3′ shortening with age.

C) Sequencing coverage at nucleotides -3 to 3 relative to the canonical 5′ end (position 0) of endogenous and spike-in RNAs in cortex Combo-Seq libraries. Coverage is stable with age in both endogenous and spike-in RNAs, consistent with the 5′ end position remaining stable with age.

D) Sequencing coverage at nucleotides -3 to 3 relative to the canonical 5′ end (position 0) of endogenous and spike-in RNAs in hippocampus Combo-Seq libraries. Coverage is stable with age in both endogenous and spike-in RNAs, consistent with the 5′ end position remaining stable with age.

E) Sequencing coverage at nucleotides 17-25 relative to the canonical 5′ end of endogenous -3p and -5p miRNAs in cortex Combo-Seq libraries. Coverage decreases at positions 19-22 of -3p miRNAs and positions 21-22 of -5p miRNAs between P22 and P120.
F) Sequencing coverage at nucleotides 17-25 relative to the canonical 5′ end of endogenous -3p and -5p miRNAs in hippocampus Combo-Seq libraries. Coverage decreases at positions 19-22 of -3p miRNAs and positions 21-22 of -5p miRNAs between P22 and P120.

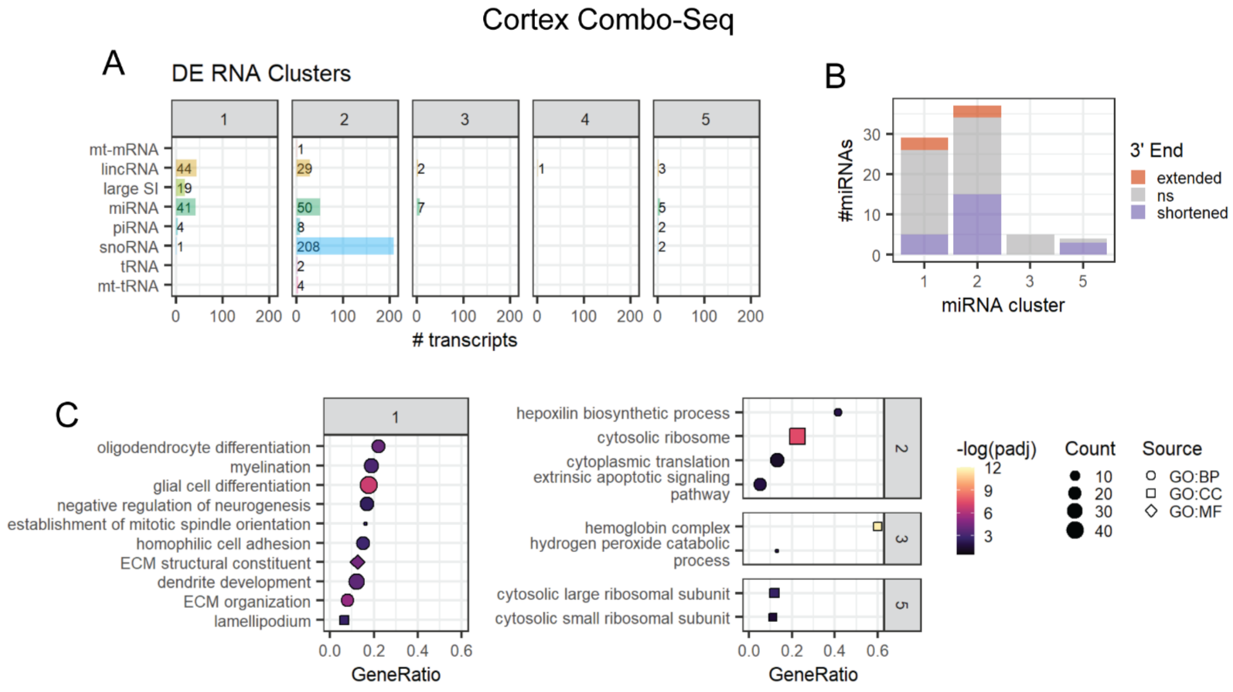


**Figure S5**

Cortex Combo-Seq results

A) DE RNAs by type and by cluster. See Figure 4B for associated expression patterns. mRNAs (not shown) constituted the majority of all DE clusters.

B) Bar graph depicting the number of miRNAs with each 3′ end type in each DE RNA cluster. Cluster 2 (RNAs that increase with age) contains the most miRNAs that undergo 3′ shortening with age.

C) GO term enrichment results for DE mRNAs by cluster. Shapes indicate term sub-ontology. BP: biological process. CC: cellular component. MF: molecular function.

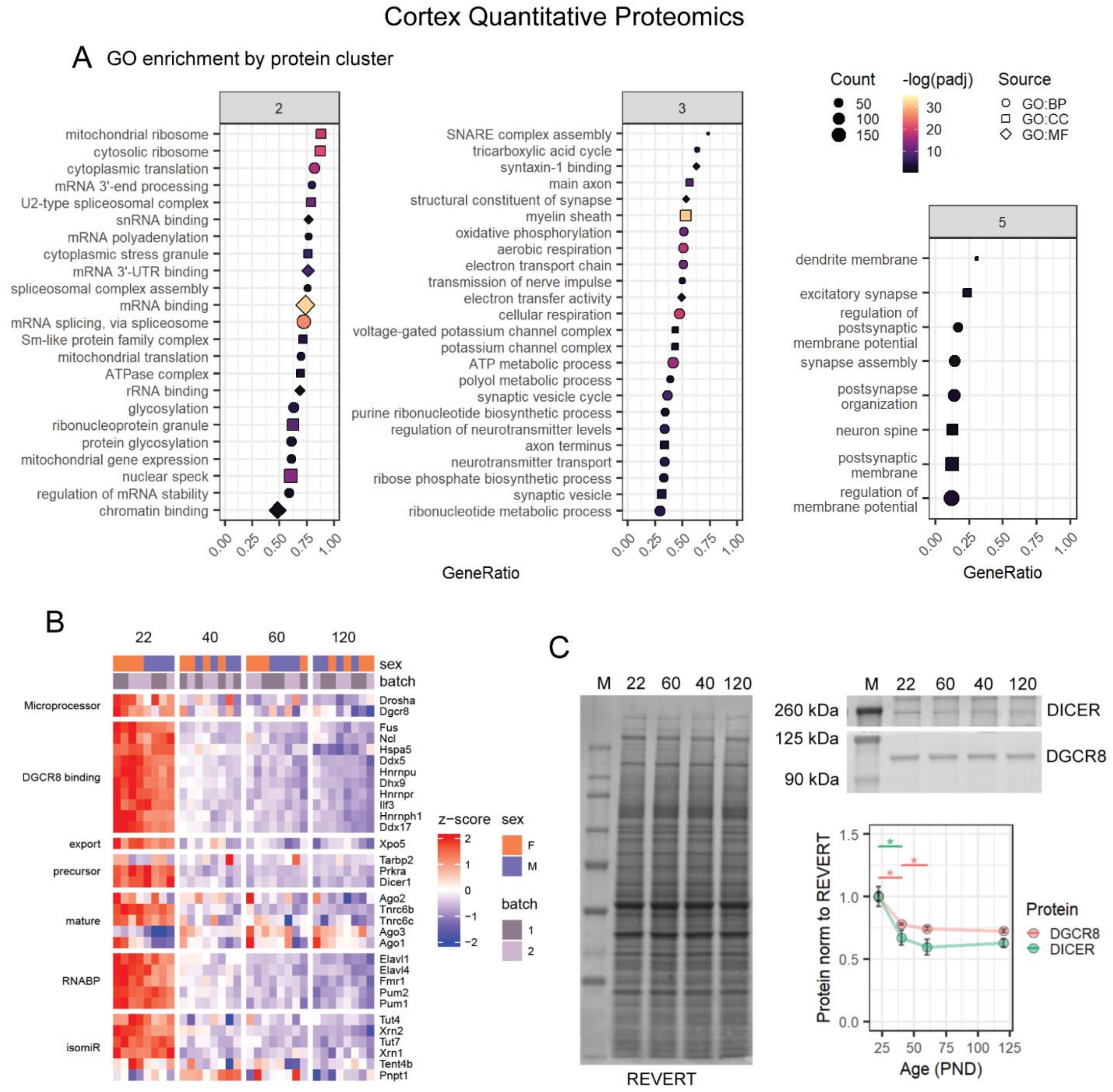


**Figure S6**

Cortex quantitative proteomics results

A) GO term enrichment results for DE proteins by cluster. See Figure 4E for associated expression patterns.

B) Heatmap depicting expression of proteins that regulate miRNA synthesis (Microprocessor, DGCR8 binding, export, precursor), function (mature, RNABP), and sequence and/or stability (isomiR).

C) Western blot validation of proteomics results that DICER and DGCR8 decrease with age. REVERT total protein stain (left) was used for data normalization. Representative blots of DICER and DGCR8 are shown in the top right, with data summarized in the bottom right. One way ANOVA: p(DICER) = 0.004, p(DGCR8) = 0.035. Holm-adjusted p-values for pairwise comparisons: *$p < 0.05$.

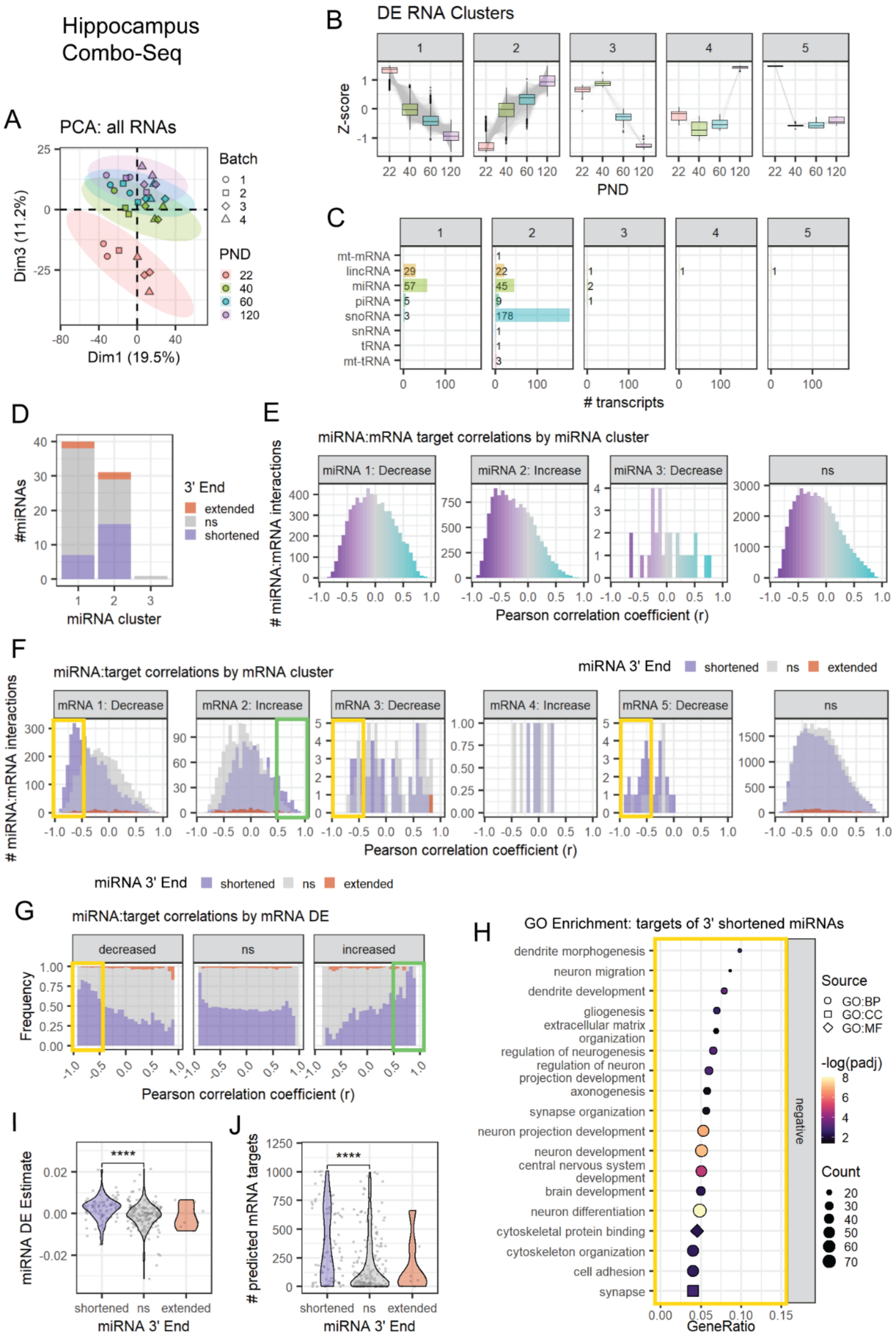
Hippocampus
Combo-Seq
A
PCA: all RNAs
Dim3 (11.2%)
Dim1 (19.5%)
Batch
1
2
3
4
PND
22
40
60
120
B
DE RNA Clusters
Z-score
PND
C
mt-mRNA
lincRNA
miRNA
piRNA
snoRNA
snRNA
tRNA
mt-tRNA
# transcripts
D
#miRNAs
miRNA cluster
3' End
extended
ns
shortened
E
miRNA:mRNA target correlations by miRNA cluster
miRNA 1: Decrease
miRNA 2: Increase
miRNA 3: Decrease
ns
# miRNA:mRNA interactions
Pearson correlation coefficient (r)
F
miRNA:target correlations by mRNA cluster
miRNA 3' End
shortened
ns
extended
mRNA 1: Decrease
mRNA 2: Increase
mRNA 3: Decrease
mRNA 4: Increase
mRNA 5: Decrease
ns
# miRNA:mRNA interactions
Pearson correlation coefficient (r)
miRNA 3' End
shortened
ns
extended
G
miRNA:target correlations by mRNA DE
decreased
ns
increased
Frequency
Pearson correlation coefficient (r)
H
GO Enrichment: targets of 3' shortened miRNAs
dendrite morphogenesis
neuron migration
dendrite development
gliogenesis
extracellular matrix organization
regulation of neurogenesis
regulation of neuron projection development
axonogenesis
synapse organization
neuron projection development
neuron development
central nervous system development
brain development
neuron differentiation
cytoskeletal protein binding
cytoskeleton organization
cell adhesion
synapse
negative
GeneRatio
Source
GO:BP
GO:CC
GO:MF
-log(padj)
Count
I
miRNA DE Estimate
shortened
ns
extended
miRNA 3' End
J
# predicted mRNA targets
shortened
ns
extended
miRNA 3' End

**Figure S7**

MiRNAs that undergo 3′ shortening with age exhibit stronger correlations with mRNA target levels in hippocampus

A) PCA was performed on count data for all RNAs in hippocampus Combo-Seq dataset, including mRNAs and miRNAs. QuagmiR-mapped counts were used for miRNAs. All other count data came from the exceRpt pipeline. The third dimension exhibits an association with sample age.
B) Clusters representing DE patterns for RNAs in hippocampus.
C) DE RNAs by type and by cluster. mRNAs (not shown) composed the majority of all DE clusters.
D) Bar graph depicting the number of miRNAs with each 3′ end type in each DE RNA cluster. Cluster 2 (RNAs that increase with age) contains the most miRNAs that undergo 3′ shortening with age.
E) Pearson correlation coefficient (r) distributions for miRNAs and their predicted mRNA targets, with panels representing the corresponding miRNA cluster. Y-axis reflects the number of interactions with each r value.
F) Pearson correlation coefficient (r) distributions for miRNAs and their predicted targets, with panels representing the mRNA target cluster. Y-axis reflects the number of predicted miRNA:target interaction pairs at each r value. Bar color reflects each miRNA’s 3′ end dynamics with age.
G) Pearson correlation analysis organized by mRNA target DE results. Y-axis indicates the relative frequency of targeting by miRNAs that have shortened, extended, or stable (ns) 3′ ends with age. Bar color reflects miRNA 3′ end dynamics with age.
H) GO term enrichment analysis results for mRNA targets that decreased with age and exhibited negative correlations ($r < -0.5$, orange box in F and G). No enrichment was observed for targets that increased with age and exhibited positive correlations. Shapes indicate term sub-ontology. BP: biological process. CC: cellular component. MF: molecular function.
I) Violin plot representing the relationship between miRNA 3′ end dynamics with age and the miRNA’s DE Estimate, which summarizes the change in miRNA levels with age. Positive DE Estimate values reflect miRNAs that increase with age, while negative values reflect miRNAs that decrease with age. Each point represents one miRNA. Kruskal-Wallis: $p = 1e-05$. Wilcox test for pairwise comparisons: ****$p < 0.0001$.
J) Violin plot representing the relationship between miRNA 3′ end dynamics with age and the number of predicted mRNA targets detected for each miRNA. Each point represents one miRNA. Kruskal-Wallis: $p = 5.4e-06$. Wilcox test for pairwise comparisons: ****$p < 0.0001$.

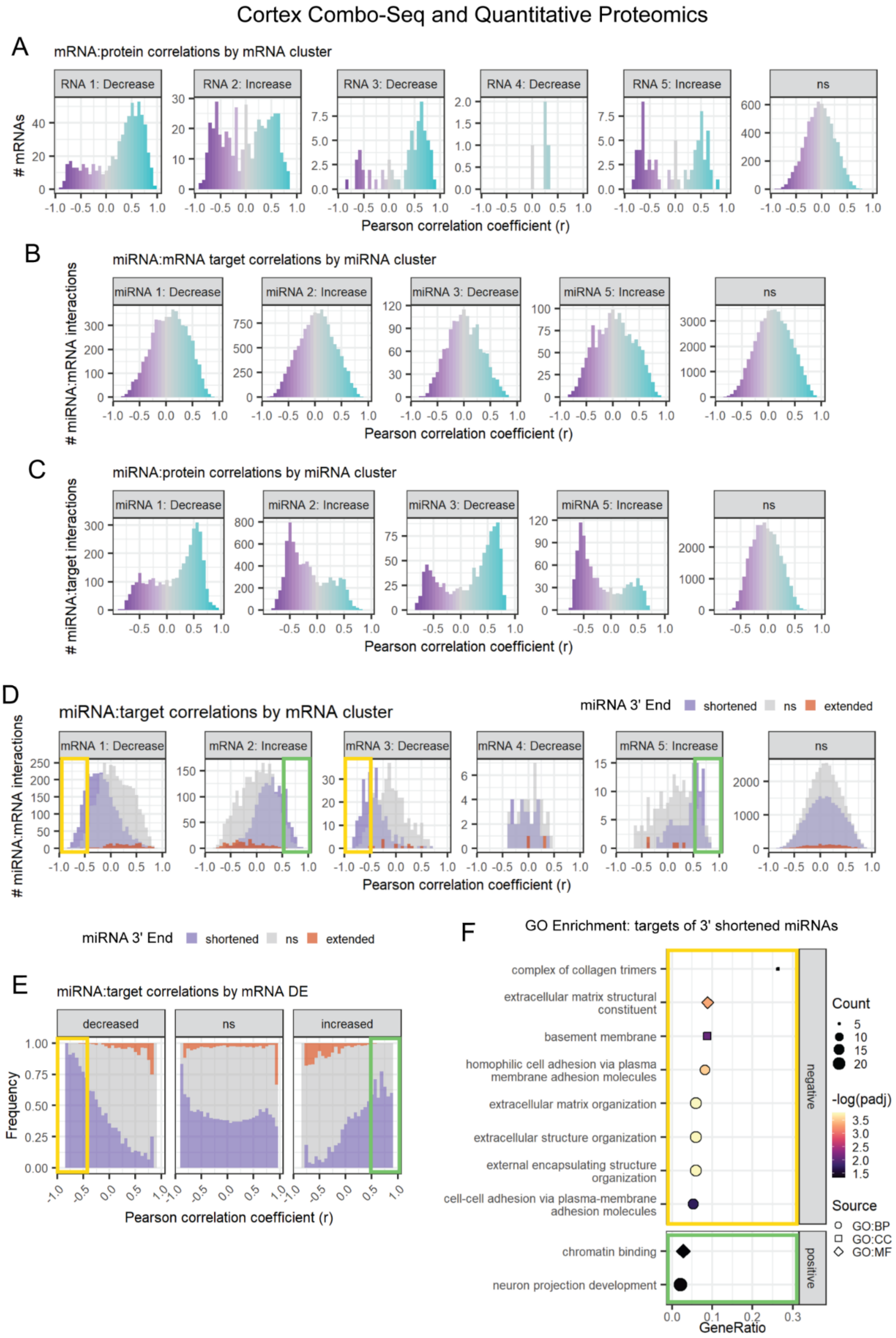

Cortex Combo-Seq and Quantitative Proteomics
A
mRNA:protein correlations by mRNA cluster
RNA 1: Decrease
RNA 2: Increase
RNA 3: Decrease
RNA 4: Decrease
RNA 5: Increase
ns
# mRNAs
Pearson correlation coefficient (r)
B
miRNA:mRNA target correlations by miRNA cluster
miRNA 1: Decrease
miRNA 2: Increase
miRNA 3: Decrease
miRNA 5: Increase
ns
# miRNA:mRNA interactions
Pearson correlation coefficient (r)
C
miRNA:protein correlations by miRNA cluster
miRNA 1: Decrease
miRNA 2: Increase
miRNA 3: Decrease
miRNA 5: Increase
ns
# miRNA:target interactions
Pearson correlation coefficient (r)
D
miRNA:target correlations by mRNA cluster
miRNA 3' End
shortened
ns
extended
mRNA 1: Decrease
mRNA 2: Increase
mRNA 3: Decrease
mRNA 4: Decrease
mRNA 5: Increase
ns
# miRNA:mRNA interactions
Pearson correlation coefficient (r)
miRNA 3' End
shortened
ns
extended
E
miRNA:target correlations by mRNA DE
decreased
ns
increased
Frequency
Pearson correlation coefficient (r)
F
GO Enrichment: targets of 3' shortened miRNAs
complex of collagen trimers
extracellular matrix structural constituent
basement membrane
homophilic cell adhesion via plasma membrane adhesion molecules
extracellular matrix organization
extracellular structure organization
external encapsulating structure organization
cell-cell adhesion via plasma-membrane adhesion molecules
chromatin binding
neuron projection development
negative
positive
GeneRatio
Count
-log(padj)
Source
GO:BP
GO:CC
GO:MF


**Figure S8**

MiRNAs that undergo 3′ shortening with age exhibit stronger correlations with mRNA target levels in cortex.

A) Pearson correlation coefficient (r) distributions for mRNAs and their encoded proteins in cortex, with data organized by mRNA cluster. Y-axis reflects the number of mRNA:protein pairs at each r value.
B) Pearson correlation coefficient (r) distributions for miRNAs and their predicted mRNA targets, with data organized by miRNA cluster. Y-axis reflects the number of miRNA:target pairs at each r value.
C) Pearson correlation coefficient (r) distributions for miRNAs and proteins encoded by their predicted mRNA targets, with data organized by miRNA cluster. Y-axis reflects the number of miRNA:target pairs at each r value.
D) Pearson correlation coefficient (r) distributions for miRNAs and their predicted targets, with panels representing the mRNA target cluster. Y-axis reflects the number of predicted miRNA:target interaction pairs at each r value. Bar color reflects each miRNA's 3′ end dynamics with age.
E) Pearson correlation analysis organized by mRNA target DE results. Y-axis indicates the relative frequency of targeting by miRNAs that have shortened, extended, or stable (ns) 3′ ends with age. Bar color reflects miRNA 3′ end dynamics with age.
F) GO term enrichment analysis results for mRNA targets that decreased with age and exhibited negative correlations ($r < -0.5$, orange box in F and G) or increased with age and exhibited positive correlations ($r > 0.5$, green box in F and G) with miRNAs that underwent 3′ shortening. Shapes indicate term sub-ontology. BP: biological process. CC: cellular component. MF: molecular function.

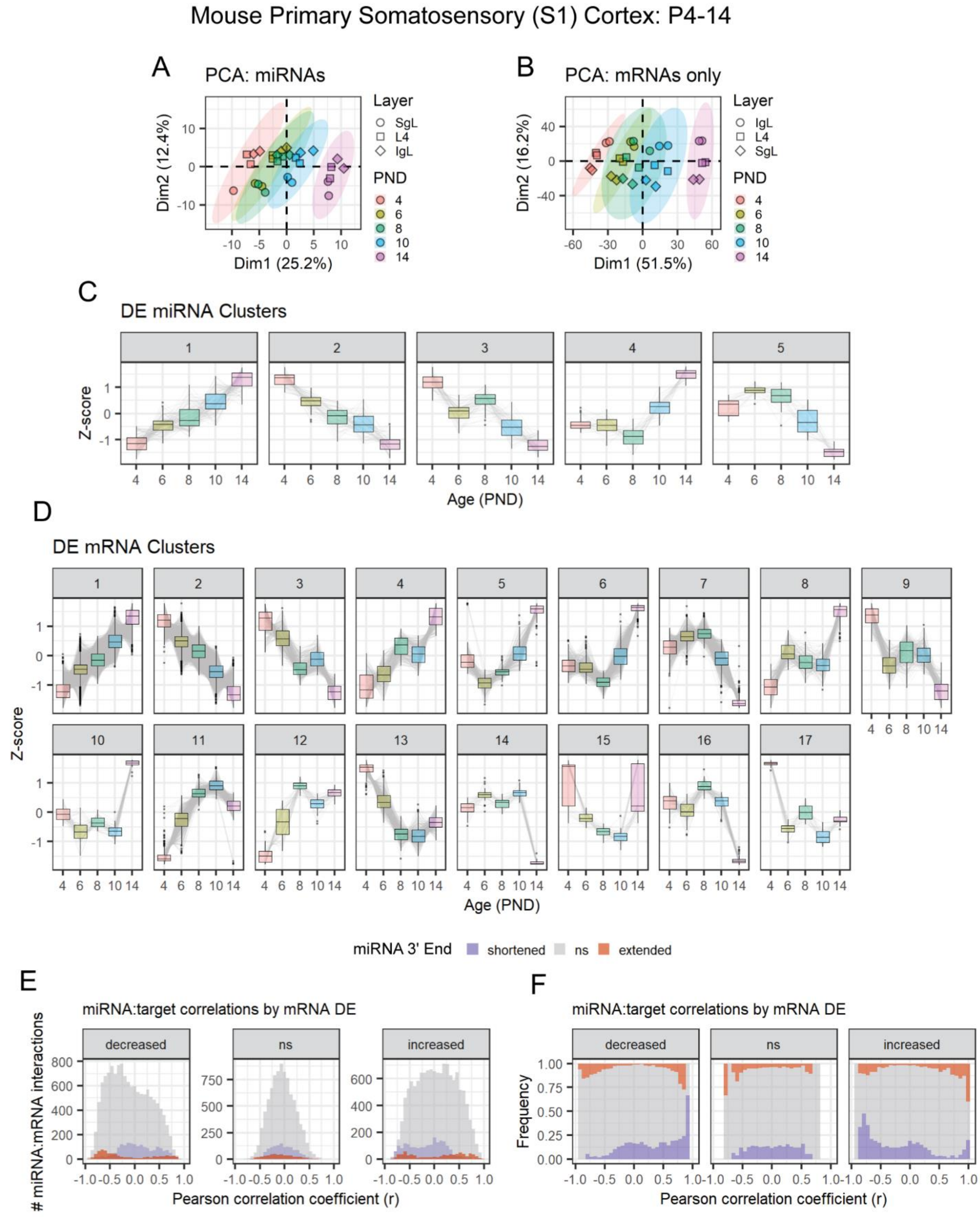


**Figure S9**

DE and correlation analysis for miRNAs and mRNAs in S1 cortex between P4 and P14

A) PCA was performed on miRNA-seq count data derived from mouse S1 cortex ages P4-14. Samples clustered by age in dimension 1 and by cortical layer in dimension 2.

B) PCA was performed on mRNA-seq count data derived from mouse S1 cortex ages P4-14. Samples clustered by age in dimension 1 and by cortical layer in dimension 2. No outliers were identified.
C) Clusters representing DE patterns for miRNAs.
D) Clusters representing DE patterns for mRNAs.
E) Pearson correlation coefficient (r) distributions for miRNAs and their predicted targets, with panels organized by mRNA DE results. Y-axis reflects the number of predicted miRNA:target interaction pairs at each r value. Bar color reflects each miRNA's 3′ end dynamics with age.
F) Pearson correlation analysis organized by mRNA target DE results. Y-axis indicates the relative frequency of targeting by miRNAs that have shortened, extended, or stable (ns) 3′ ends with age. Bar color reflects miRNA 3′ end dynamics with age.

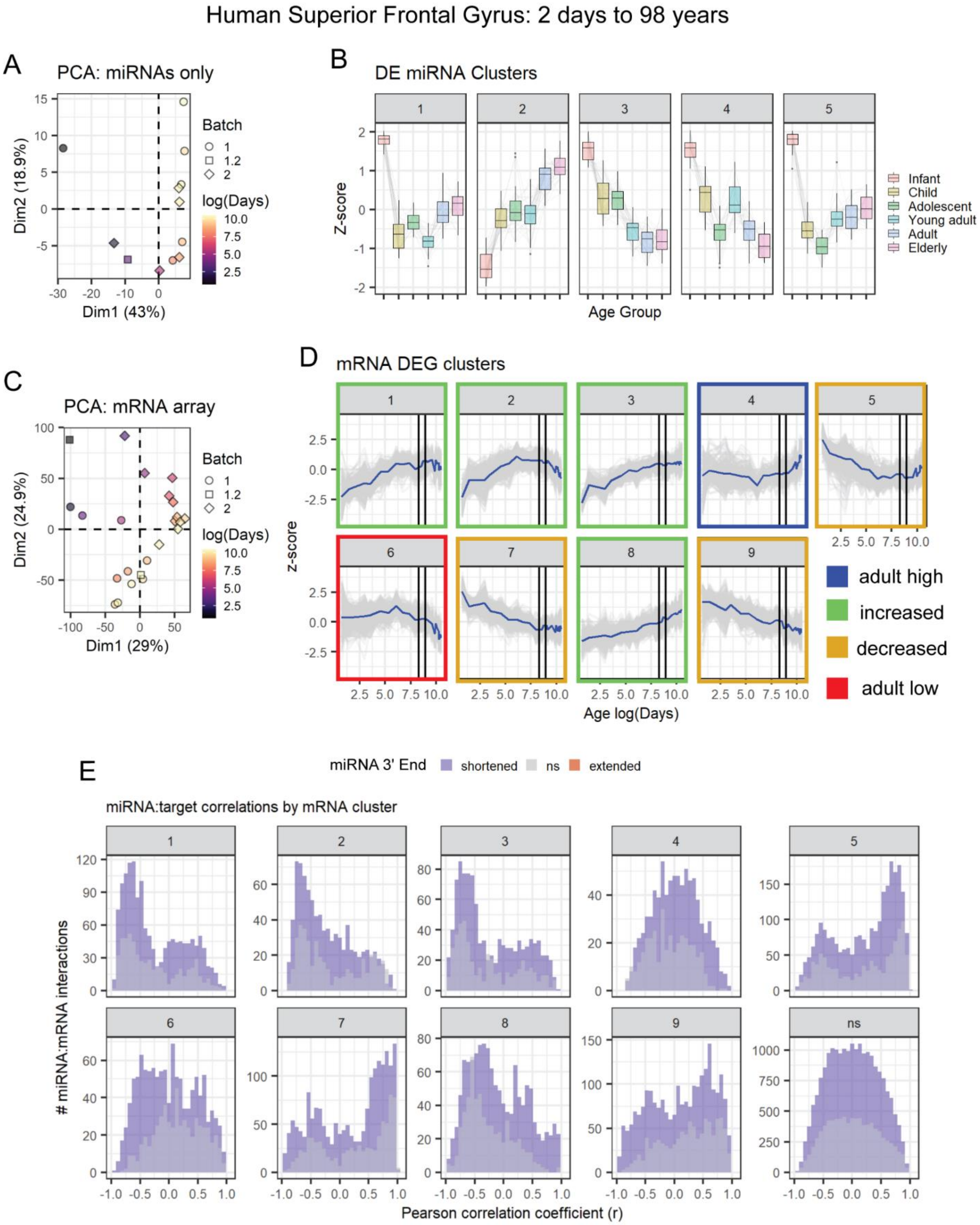


**Figure S10**

DE and correlation analysis for miRNAs and mRNAs in human superior frontal gyrus from subjects between 2 days and 98 years of age

A) PCA was performed on miRNA-seq count data. Samples approximately aligned by subject age.
B) Clusters representing DE patterns for miRNAs.
C) PCA was performed on mRNA microarray expression data. Samples approximately arranged by subject age.
D) Clusters representing DE patterns for mRNAs. Adolescent age range is indicated by black bars. Panels are color coded by their assignment to mRNA DE groups in Figure 8G-H.
E) Pearson correlation analysis was performed for miRNAs and their predicted mRNA targets. Data are organized in histograms by mRNA cluster. Y-axis reflects the number of predicted miRNA:target interaction pairs at each Pearson correlation coefficient, r, value. Bar color reflects each miRNA's 3′ end dynamics with age.
